# A metal ions-mediated natural small molecules carrier-free injectable hydrogel achieving laser-mediated photo-Fenton-like anticancer therapy by synergy apoptosis/cuproptosis/anti-inflammation

**DOI:** 10.1016/j.bioactmat.2023.06.018

**Published:** 2023-07-05

**Authors:** Wenmin Pi, Linying Wu, Jihui Lu, Xiaoyu Lin, Xuemei Huang, Zhijia Wang, Zhihua Yuan, Hailing Qiu, Jianglan Zhang, Haimin Lei, Penglong Wang

**Affiliations:** a School of Chinese Pharmacy, Beijing University of Chinese Medicine, Beijing, 102488, China; b School of Traditional Chinese Medicine, Beijing University of Chinese Medicine, Beijing, 102488, China

**Keywords:** Natural small molecular ternary carrier-free hydrogel, Tumor microenvironment, Cuproptosis, Laser-mediated photo-fenton-like reaction

## Abstract

Tumor microenvironment (TME) plays an important role in the tumorigenesis, proliferation, invasion and metastasis. Thereby developing synergistic anticancer strategies with multiple mechanisms are urgent. Copper is widely used in the treatment of tumor chemodynamic therapy (CDT) due to its excellent laser-mediated photo-Fenton-like reaction. Additionally, copper can induce cell death through cuproptosis, which is a new modality different from the known death mechanisms and has great promise in tumor treatment. Herein, we report a natural small molecules carrier-free injectable hydrogel (NCTD Gel) consisted of Cu^2+^-mediated self-assembled glycyrrhizic acid (GA) and norcantharidin (NCTD), which are mainly governed by coordination and hydrogen bonds. Under 808 nm laser irradiation, NCTD Gel can produce reactive oxygen species (ROS), consume glutathione (GSH) and overcome hypoxia in TME, leading to synergistically regulate TME via apoptosis, cuproptosis and anti-inflammation. In addition, NCTD Gel's CDT display high selectivity and good biocompatibility as it relies on the weak acidity and H_2_O_2_ overexpression of TME. Notably, NCTD Gel's components are originated from clinical agents and its preparation process is easy, green and economical, without any excipients. This study provides a new carrier-free hydrogel synergistic antitumor strategy, which has a good prospect in industrial production and clinical transformation.

## Highlights

• A copper ion-mediated natural small molecules carrier-free injectable hydrogel (NCTD Gel) has been developed.
• NCTD Gel with laser-mediated photo-Fenton-like reaction can be used in the treatment of tumor chemodynamic therapy (CDT).
• NCTD Gel regulates tumor microenvironment by synergy apoptosis, cuproptosis and anti-inflammation.

## Graphical abstract

**Figure:**
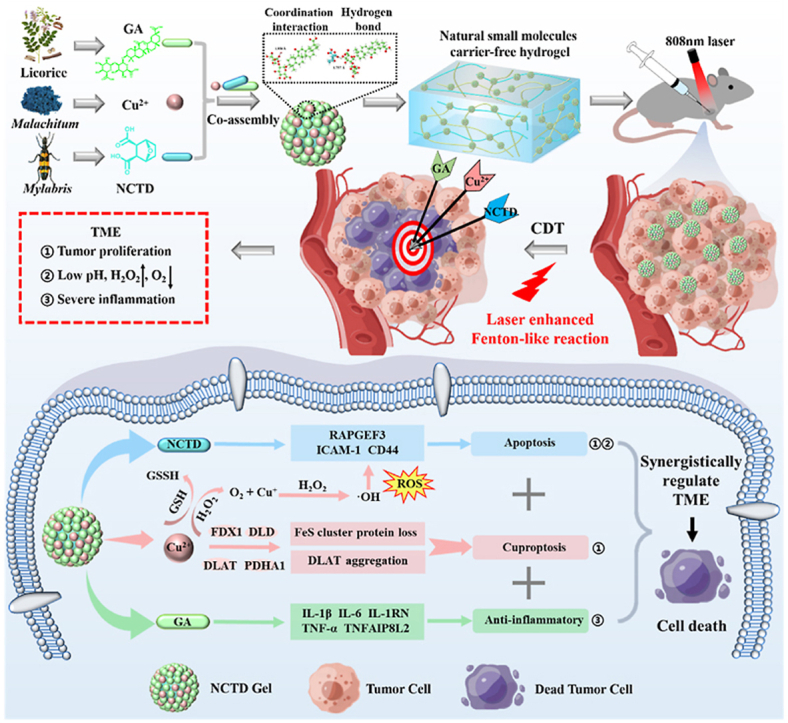

## 1 Introduction

Cancer is a leading cause of death in the world, which were 19.3 million new cases and nearly 10 million deaths in 2020 [1,2]. Currently, surgery resection and chemotherapy are the main treatment modalities for cancer in clinic [3,4]. Although chemotherapeutic agents are effective in killing tumor cells, the drawbacks of drug resistance, low targeting and serious adverse reactions limit their clinical application [5]. Except the above-mentioned barriers of tumor treatment, the tumor microenvironment (TME) is complicated and has played a crucial role in tumorigenesis, invasion, metastasis and recurrence, which is the major challenges in cancer treatment [6]. TME originated from the “seed and soil” hypothesis of tumor metastasis in 1889 by British physician Stephen Paget [7]. Recently, more and more studies have begun to formulate treatment strategies for the characteristics of the TME, including weak acidity, H_2_O_2_ overexpression, hypoxia and severe inflammation reaction [8,9]. Among them, anti-inflammatory modulator has been used for TME regulation achieving satisfactory synergistic therapeutic effect in clinic [10]. In the meanwhile, several metal ions with Fenton/Fenton-like reaction are widely used in antitumor chemodynamic therapy (CDT) [11]. CDT induces apoptosis relying on production reactive oxygen species (ROS) such as hydroxyl radicals (·OH) and superoxide anions (·O^2−^) in the TME [12]. In particular, near-infrared laser irradiation can improve ROS generation efficiency in Fenton-like reaction to enhance antitumor effect of CDT [13]. CDT and inflammatory regulation denote a promising emerging strategy for tumor treatment due to its high specificity, low side effects and noninvasiveness [14].

Recently, many bionanomaterials and hydrogels have been developed for CDT [15,16]. Although bionanomaterials possess the active or passive tumor-targeting effect in systemic administration, recent studies indicate that only a tiny part of nanoparticles (0.7%, median) can reach tumor tissue after intravenous injection, potentially causing unnecessary toxicities on normal tissues and organs [17,18]. Meanwhile, antitumor hydrogels are of increasing interest to scholars. Hydrogel is more like a warehouse due to its characteristics of three-dimensional networks and various physical properties, which can continuously release drugs to the target site, with minimal side effects on normal tissues and organs [19]. In particular, injectable hydrogels show great prospects for the personalized medicine due to their advantages of local targeting and minimally invasive drug delivery [20]. Until now most of the developed hydrogels are used as carriers for encapsulating various drugs [21]. And they need the participation of polymer materials such as hyaluronic acid [22], alginate [23] and chitosan [24]. Nevertheless, the inflammatory regulatory efficacy of these high molecular carriers is usually weaker in TME than small molecular anti-inflammatory agents such as glycyrrhizic acid (GA). In addition, the involvement of carriers may lead to low drug loading, poor biodegradability and biocompatibility, as well as potential side effects, which are the main reasons hinder the industrial production and clinical transformation [25]. These unavoidable obstacles prompt an urgent need to develop synergistic anticancer strategies with different mechanisms of action to provide an alternative pathway for CDT.

**Fig. 1:**
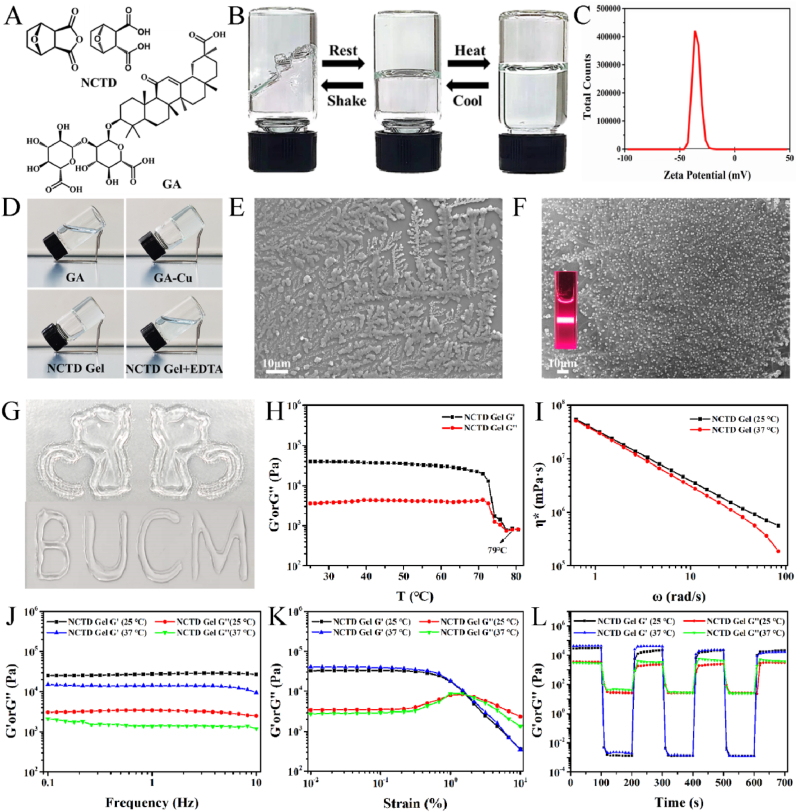
Characteristics of NCTD Gel. **A** Chemical structure of NCTD and GA. **B** Reversible gel-sol transitions of NCTD Gel triggered by shear stress and temperature. **C** Zeta potential of NCTD Gel. **D** Macrograph of GA alone, and with addition of Cu^2+^, NCTD and EDTA in turn. **E** SEM image of GA-Cu Gel. **F** Tyndall effect and SEM image of NCTD Gel. **G** Photographs of the injectability and moldability of NCTD Gel. **H** Rheological analysis of temperature of NCTD Gel. **I** Viscosity measurement of NCTD Gel, measured at 0.1% strain. **J** Dynamic frequency sweep of the NCTD Gel, measured at 0.1% strain. **K** Strain-dependent oscillatory shear rheology of NCTD Gel, at the frequency of 1 Hz. **L** Step-strain measurements of NCTD Gel at low strain (0.1%) and high strain (300%) at 1 Hz frequency.

To solve the above challenges, we selected three kinds of strategies to coordinate the regulation of TME to achieve the multiple antitumor therapy. Firstly, copper is an essential trace element for all organisms, and it is also the main component of traditional medicine *Malachitum*, which has been used to antitumor since the Ming Dynasty *Compendium of Materia Medica*. Importantly, copper has the Fenton-like reaction as transition metal element, which has attracted much attention in CDT of cancer [26]. Unlike the Fenton effect of Fe^2+^, which works efficiently under acidic condition (pH 2.0–4.5) [11]. Cu^2+^ has a wide pH working range, so that it can exert Fenton-like reaction under weak acidic and neutral conditions. Considering that the TME is weakly acidic (pH 6.5–7.0), Cu^2+^ can be more promising in CDT than Fe^2+^ [27]. Copper ions have convertible valences (Cu^2+^ and Cu^+^) that can act as active center in Fenton-like reaction. Cu^2+^ can work like catalase to catalyze the generation of oxygen from the overexpressed H_2_O_2_, which combat hypoxia in TME; Cu^+^ can catalyze H_2_O_2_ to produce hydroxyl radical and other highly toxic reactive oxygen species. Meanwhile, Cu^2+^ reacts with GSH to generate GSSH, which protects the generated ROS and induces apoptosis of tumor cells [28]. Most importantly, copper and other transition metal coordination complexes have well laser-induced absorption properties in the near-infrared region (808 nm), which have great development potential for CDT [29,30]. Recently, researchers have identified a form of regulatory tumor cell death induced by excess copper-cuproptosis [31,32]. Cuproptosis is a new cell death mechanism different from ferroptosis and apoptosis, which opens a new pathway for the development of cancer treatment. Secondly, GA is a main active ingredient of licorice (Gancao), which has promising anti-inflammatory effect [33]. As shown in Fig. 1A, GA is an amphiphilic molecule in terms of the chemical structure and can be self-assembled into hydrogel in physiological environment [34]. GA hydrogel has good biocompatibility and biodegradability, which is widely used in the field of materials science. As recently reported, Zn^2+^ and other polyvalent metal ions can promote the self-assembly of GA at lower concentrations [21]. Moreover, GA has Cox-2-mediated anti-inflammatory responses and can act as HMGB1-TLR4 axis inhibitor to inhibit the proliferation, invasion and metastasis in TME [[35], [36], [37]]. Thirdly, norcantharidin is a demethylated derivative of cantharidin, the main anticancer component of traditional Chinese medicine cantharides (Banmao), which is an effective anticancer drug in clinical treatment of liver cancer, stomach cancer and so on [38,39]. Especially, the combination of cantharides and licorice is used to antitumor in Chinese patent medicine Fufang Banmao capsule. Norcantharidin as well as its diacid form (hereinafter referred as NCTD) is shown in Fig. 1A, which have strong inhibitory and induce apoptosis effects against a broad range of cancer cells in clinic [40]. Unfortunately, NCTD has poor water solubility, short half-life in blood, and low tumor-targeting efficiency, which significantly hinder its usage [41].

**Scheme 1:**
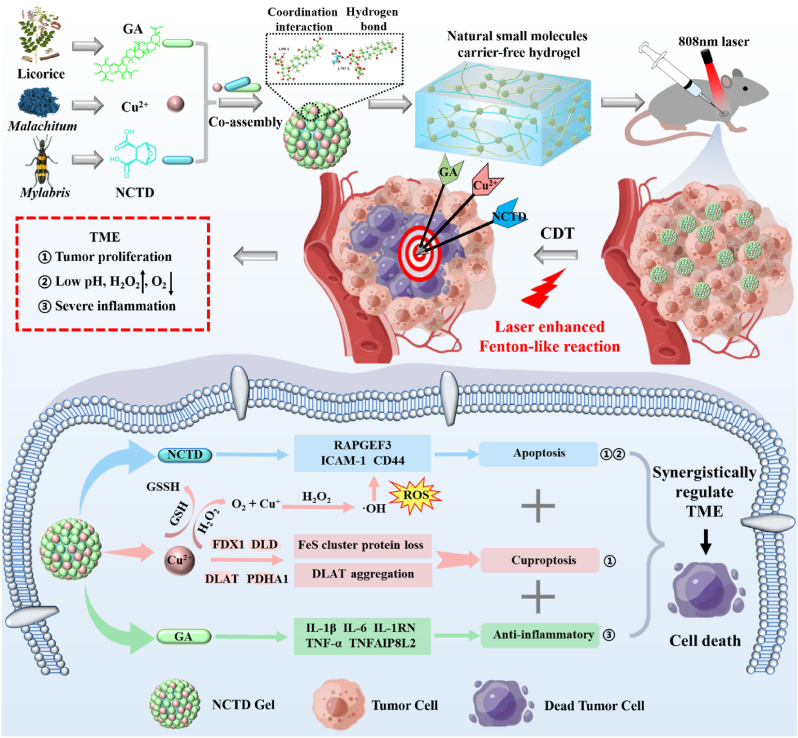
Schematic illustration of NCTD Gel's self-assembly mechanism and synergistically regulate the tumor microenvironment via apoptosis, cuproptosis and anti-inflammation.

Based on our previous studies on constructing the carrier-free self-assemblies [[42], [43], [44]], a copper ion-mediated natural small molecules carrier-free injectable hydrogel with CDT characters is developed to synergistically antitumor effect through apoptosis, cuproptosis and anti-inflammation to achieve the regulation of TME (Scheme 1). This ternary hydrogel (NCTD Gel) is mainly governed by coordination interaction and hydrogen bond between GA, Cu^2+^ and NCTD, which has well mechanical properties and display great performance in biomedical fields for the targeted drug delivery and the controlled release. In particular, NCTD Gel exerts better antitumor efficacy and biocompatibility than free NCTD. And under the laser, the antitumor effect of the hydrogel is further enhanced due to the laser-mediated photo-Fenton-like reaction. Furthermore, the mechanism of NCTD Gel's superior antitumor efficacy is demonstrated that it can regulate cuproptosis (FDX1, DLD, DLAT, PDHA1), inflammation (IL-1β, TNFAIP8L2, IL-1RN, IL-6, TNF-α), apoptosis and metastasis (CD44, ICAM-1 and RAPGEF3) related genes and proteins. Most importantly, the content of H_2_O_2_ in TME is much higher than that of normal tissues. The ternary carrier-free hydrogel's CDT is highly selective and safe as it relies on the weak acidity and H_2_O_2_ overexpression of TME. NCTD Gel not only circumvents the costly and the complicated procedures for injectable antitumor hydrogel, but also its components are originated from the clinical agents without modification, demonstrating a novel and promising tumor treatment in CDT and potential clinical transformation.

## 2 Material and methods

### 2.1 Preparation of NCTD Gel

One-pot preparation procedure was used to prepare NCTD Gel. In brief, GA and NCTD were dissolved in deionized water and heated at 80 °C to obtain aqueous solutions of GA and NCTD, respectively. CuCl_2_·2H_2_O was dissolved in deionized water to obtain CuCl_2_ aqueous solution. Subsequently, GA, CuCl_2_ and NCTD aqueous solutions were mixed well by heating to 80 °C for 10 min at a molar ratio of 2:1:2. After cooling to room temperature, GA, Cu^2+^ and NCTD could rapidly co-assembly to form NCTD Gel. The gelation of hydrogel was evaluated by the tube inversion test. If no visible fluidity was observed when the tube was inverted for 1 min, the formation of the hydrogel was confirmed.

### 2.2 Characterization of NCTD Gel

Zeta potential was detected by Malvern Zetasizer Nano ZS (Zetasizer Nano ZS 90, Malvern Instrument, UK) at 25 °C. The morphological of hydrogel was recorded by scanning electron microscopy (SEM, ZEISS-SUPRA55, Germany). Rheology tests were performed on the rheometer (MCR 302, Aaton paar, Austria) to investigate mechanical properties and stability of NCTD Gel. In order to explore self-assembly mechanism of NCTD Gel, thermodynamic mechanism was recorded on a NANO ITC (TA, USA). UV–vis spectra were recorded on a UV–vis spectrophotometer (HITACHI UH5300, Japan) with the range of 200–400 nm. FT-IR spectra were recorded on a Fourier transform infrared spectrometer (NicoletiS10, Thermo, USA) with the range of 4000-400 cm^−1^. CD spectra were obtained by using Chirascan V100 (Applied Photophysics, UK) with the range of 200–400 nm. ^1^H-NMR (Avance IIIHD 400 MHz spectrometer, Bruker, America) spectra were used to ensure the formation mechanism between GA and NCTD. MD simulation was performed by GROMACS 2019.6 software to reveal the self-assembly mechanism of NCTD Gel.

### 2.3 Detection of GSH content

0.5 mL GSH aqueous solution (20 mM) was mixed with 0.5 mL deionized water, Cu^2+^ (0.5, 1 mM) or NCTD Gel (1, 2 mM) for 30 min, respectively. Taken 20 μL solution after reacted, added 140 μL reagent Ⅱ and 40 μL reagent Ⅲ of GSH assay kit in turn. After mixing and maintaining for 2 min, measured the absorbance at 412 nm with a microplate reader (MK3, Thermo, USA). To detect the GSH content of cells, collected the cells firstly. And then washed them twice with PBS, added reagent Ⅰ of GSH assay kit to resuspend the cells. Sonicated at 200 W for 3 s and then stopped for 10 s, repeated 30 times. Finally, centrifuged at 8 000 g for 10 min and collected the supernatant for detection. As for tumor tissues, which need to add reagent Ⅰ and fully grind with a tissue homogenizer, and then detected according to the above method.

### 2.4 Detection of ∙OH content

Cu^2+^ (1 mM, 1200 μL) or NCTD Gel (2 mM, 1200 μL) mixed with H_2_O_2_ solution (1 M, 50 μL). A GSH solution (50 mM, 100 μL) and an MB solution (1 mg/mL, 10 μL) were successively added. After irradiation with 808 nm laser (1.0 W cm^−2^, 30 min), the solution was measured by UV–vis spectrophotometer with the range of 550–750 nm.

### 2.5 In vitro cytotoxicity assay

HepG2 cells were cultured in 96-well plate. And then a different concentration of GA-Cu, free NCTD and NCTD Gel were added into the 96-well plate when cells were adhered after 24 h incubation. Thereafter, MTT was added after 72 h to detect the cell viability. Following, the cell viability of NCTD Gel in response to H_2_O_2_ and GSH stimuli, laser irradiation, and pH changes were detected by MTT method. Meanwhile, fluorescence microscope (xioZoom V16, Nikon, Japan) was used to observe the cellular morphology changes and DAPI staining results.

### 2.6 Determination of release properties *in vitro*

The normal microenvironment was pH = 7.4 PBS buffer. The tumor microenvironment was pH = 6.5 PBS buffer + H_2_O_2_ + GSH and the concentration of H_2_O_2_ and GSH was 1 mM. An 808 nm laser was added to the tumor microenvironment to verify efficacy. With PBS buffer as the release medium under the above three conditions, 1 mL NCTD Gel was removed to the dialysis bag with the interception molecular weight of 3000, and 1 mL PBS was added for dispersion. The dialysis bag was placed into 19 mL PBS buffer and released dynamically at 25 °C under 500 r·min^−1^. At 1, 2, 4, 6, 8, 10, 12, 24 h, 200 μL of dialysis solution was removed (and the same amount of releasing medium was added), respectively. The content of GA was determined by High performance Liquid Chromatography (Agilent, Agilent Technologies Co., LTD. USA), and the cumulative release of GA in NCTD Gel was calculated. The results were repeated three times.

Chromatographic conditions: AQ-C_18_ column (250 mm × 4.6 mm, 5 μm, Agilent) was used. The mobile phase consisted of (A) acetonitrile and (B) 0.1% (v/v) aqueous phosphoric acid solution. The gradient elution conditions were 0–5 min, 60% A. The flow rate was 1 mL/min and the injection volume was 5 μL. The detection wavelength was 250 nm and the column temperature was 25 °C.

### 2.7 In vitro apoptosis detection

The apoptosis detection was performed with Annexin V-FITC apoptosis detection kit (Beyotime, Shanghai, China). HepG2 cells were cultured in 6-well plate. After 24 h incubation, GA-Cu, free NCTD and NCTD Gel (32 μg/mL) were added into each well. In the meanwhile, H_2_O_2_ or 808 nm laser irradiation (1.0 W∙cm^−2^, 1 min) was added into NCTD Gel. Afterwards, HepG2 cells were collected after 48 h incubation and washed twice with cold PBS (4 °C). And then centrifuged at 2400 rpm for 10 min to obtain cells. Following, cells were resuspended in 100 μL binding buffer composed of 5 μL Annexin V-FITC and 5 μL PI for 10 min in the dark. Centrifuged again and resuspended in 100 μL binding buffer to analyze by flow cytometry (BD FACSCanto II, New Jersey, USA).

### 2.8 In vitro ROS detection

The ROS detection was performed with reactive oxygen species assay kit (Beyotime, Shanghai, China). HepG2 cells were cultured in 6-well plate. After 24 h incubation, GA-Cu, free NCTD and NCTD Gel (32 μg/mL) were added into each well. In the meanwhile, H_2_O_2_ or 808 nm laser irradiation (1.0 W cm^−2^, 1 min) was added into NCTD Gel. Afterwards, HepG2 cells were collected after 12 h incubation. The cells were resuspended in DCFH-DA (10 μM) and incubated for 20 min. After that, washed three times with DMEM and detected by flow cytometry (BD FACSCanto II, New Jersey, USA) finally.

### 2.9 In vitro cellular O_2_ measurement

Hepa 1-6 cells were cultured in the 6-well plate at a concentration of 3 × 10^5^ per well. After incubation for 12 h, the cells were administered at a concentration of 32 μg/mL. After 1 h of administration, RDPP was added and incubated for another 3 h. Then, CLSM was used to capture the fluorescence.

### 2.10 Animal experiments

To establish Hepa 1-6 tumor-bearing mice, the right axillary area of C57BL/6J female mice were subcutaneously injected with 100 μL Hepa 1-6 cells suspension (7.5 × 10^7^/mL). When the tumor volume reached about 100 mm^3^, mice were randomly divided into 6 groups (5 mice/group) and started to administrate drugs. Six groups were set in the experiment, including (1) Control; (2) Model; (3) GA-Cu; (4) Free NCTD; (5) NCTD Gel; (6) NCTD Gel + laser. The mice were given drugs by intratumoral injection and the injected dose of NCTD was 5 mg/kg body weight in each mouse on day 1, 3, 5, 7, 9 and 11. The volume of each administration was 25 μL. In the meanwhile, mice's body weight and tumor volume were recorded every two days. The tumor volume was measured with vernier caliper and calculated by the formula: volume = (length × width^2^)/2. At the end of experiments, mice were sacrificed by cervical dislocation and immediately excised tumors and major organs (heart, liver, spleen, lung and kidney).

### 2.11 In vivo long-time retention of NCTD Gel

After establishing the Hepa 1-6 tumor-bearing mice model according to the above method, NCTD Gel loaded with Cy7 and free Cy7 were injected through intratumorally, respectively. At different time points after injection, Xenogen IVIS Lumina imaging system (Perkin Elmer Inc.) was used to compare the retention effect of NCTD Gel and Cy7 in the tumor.

### 2.12 RNA sequencing (RNA-seq) and real-time quantitative polymerase chain reaction (qPCR) analysis

Fresh tumor tissues of model and NCTD Gel + laser groups were taken for detection. The methods were supplied in supporting information.

### 2.13 Immunohistochemistry (IHC) assay and immunofluorescence (IF) staining

Model and all treatment groups’ tumor tissues embedded in paraffin and sliced into thick sections. And then proceeded IHC assay and IF staining, methods were supplied in supporting information.

### 2.14 Hemolytic rate (RHR%) test

Hemolytic assay of GA-Cu, free NCTD and NCTD Gel were done using fresh rat blood. Water was the positive control and normal saline was the negative control. The samples were mixed with red blood cells to obtain 4% red blood cell solution. After incubation at 37 °C for 1 h, the absorbance of the supernatant at 570 nm was measured after centrifugation. Calculate the RHR (%) of each sample according to the following formula.

RHR (%) = (Asample - Asaline)/(Awater - Asaline) × 100%

When the RHR (%) of the sample was less than the internationally accepted 5%, it was considered to have blood compatibility.

### 2.15 Statistical analysis

All statistical analyses were performed on SPSS 20.0 (IBM, USA) software and the results were expressed as mean ± SD. Student's *t*-test was used to compare statistical differences between groups. *P < 0.05, **P < 0.01 and ***P < 0.001 were considered statistically significant.

## 3 Results and discussion

### 3.1 Morphological and mechanical characteristics of NCTD Gel

In this study, the NCTD Gel was formed in an aqueous solution by combination of GA, Cu^2+^ and NCTD with heating to obtain a homogeneous solution and subsequently cooled to room temperature to gain the self-assembly hydrogel. It had a uniform pale blue appearance due to the Cu^2+^. The NCTD Gel showed multi-responsiveness to external environment changes (Fig. 1B). When the concentration of NCTD Gel was 1.68 mg mL^−1^ (10 mM), either an increase in temperature to 80 °C or a severe handshake would trigger the gel-sol transition, but returned to the gel state after a few minutes at room temperature (25 °C). In addition, the zeta potential of NCTD Gel dissolved in aqueous solution was −35.17 ± 0.47 mV (Fig. 1C), indicating its stability due to electrostatic repulsion. Although the self-assembly behavior of GA has been previously reported [34], we found that Cu^2+^ could greatly promote and enhance the formation of GA Gel (Fig. S1). Furthermore, the Gel was stable, transparent and had the best plasticity under a GA/Cu^2+^/NCTD molar ratio of 2:1:2. However, with the addition of EDTA, NCTD Gel was transformed into solution by the destructive effect of EDTA's chelation with Cu^2+^, which proved the gel contained metal coordination bonds (Fig. 1D). Furthermore, the morphology of GA-Cu Gel and ternary NCTD Gel were observed by scanning electron microscopy (SEM), as presented in Fig. 1E–F. The images showed that irregular blocks existed in GA-Cu Gel while spherical particles with regular edges in NCTD Gel. The morphology change between GA-Cu Gel and ternary NCTD Gel indicated that we contrusted a copper ion-mediated ternary co-assembly system, which had been proved by the following spectrum characteristics and molecular dynamics testing.

It could be seen from Fig. 1G that NCTD Gel had good mechanical and physical stability, which made it possible to shape a variety of patterns and letters such as kittens and “BUCM” through syringe at room temperature. Additionally, the rheological behavior test was experimented to further investigate mechanical properties and stability of NCTD Gel. The temperature rheological analysis of NCTD Gel confirmed that when the temperature increased to nearly 80 °C, the loss modulus G" was larger than the storage modulus G′, indicating the change from gel state to solution state (solution state: G'< G", gel state: G'> G") (Fig. 1H). It also indicated that NCTD Gel was stable at both room temperature 25 °C and physiological temperature 37 °C. Furthermore, the viscoelastic properties of NCTD Gel were investigated by the rotated rheology measurement. As showed in Fig. 1I, the viscosity of NCTD Gel decreased along with the increase of frequency, which made it injectable by a syringe. The dynamic frequency sweep showed that the value of G′ was about 10 times larger than G′′ (Fig. 1J), confirming the stability of NCTD Gel. In addition, we also carried out the study of oscillatory shear rheology. When the oscillatory strain was large enough, the crossover points of G′ and G′′ occurred. This validated that the NCTD Gel had good shear thinning property (Fig. 1K). Afterwards, the step-strain test (Fig. 1L) was used to perform the self-healing behavior of NCTD Gel. When at a low strain of 0.1%, NCTD Gel could keep the gel state, while at a high strain of 300%, the G′ decreased rapidly and the structure of NCTD Gel was destroyed with the gel changed to a sol state. The repeated process yielded similar results to those in the first cycle, proving the rapid and complete healing ability of NCTD Gel. The self-healing ability of NCTD Gel is attributed to the dynamic metal coordination bond in NCTD Gel [45]. All results revealed that the ternary carrier-free NCTD Gel had perfect rheological properties, including viscoelastic property, injectability, stability, shear thinning property, self-healing ability and had great performance in biomedical fields for the targeted drug delivery and the controlled release. The current results were consistent with the recent reports that GA-component hydrogel had good physical and chemical properties [21] (Fig. 1).

### 3.2 Self-assembly mechanism of NCTD Gel

**Fig. 2:**
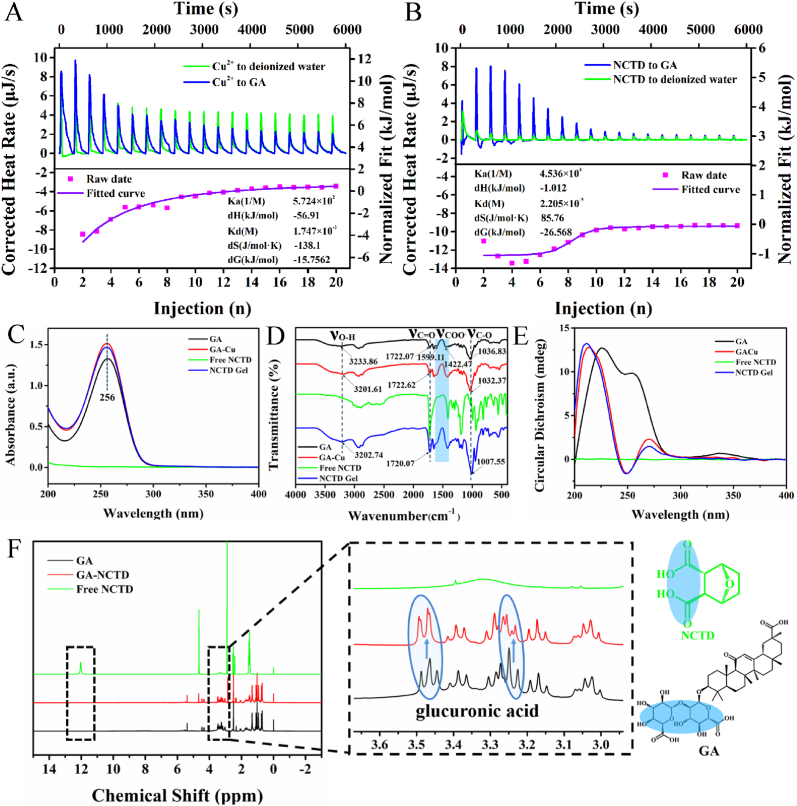
Self-assembly mechanism of NCTD Gel. Isothermal titration calorimetry (ITC) raw data fitted curves and thermodynamic parameters of **A** Cu^2+^ to GA and **B** NCTD to GA. **C** UV–vis, **D** FT-IR, and **E** CD of GA, GA-Cu, Free NCTD and NCTD Gel. **F**^1^H-NMR spectrum of GA, GA-NCTD and Free NCTD.

Revealing the molecular interactions was an effective strategy to disclose the self-assembly mechanism. The thermodynamic characters of the interaction between GA and Cu^2+^, GA and NCTD were studied by isothermal titration calorimetry (ITC). As shown in Fig. 2A and B, Cu^2+^ (40 mM) and NCTD (40 mM) titrated deionized water as blank groups, Cu^2+^ (40 mM) and NCTD (40 mM) titrated GA (2 mM) as experimental groups, respectively; the energy changes were observed and listed in Tables S1 and S2. The titration curves of both groups were upward, indicating that they were exothermic reactions. To eliminate the interference of dilution heat, the titration curve of the experimental groups was fitted after deducting the blank groups. The fitting curves and thermodynamic parameters (*K*_a_, Δ*H*, *K*_d_ and Δ*S*) of GA and Cu^2+^, GA and NCTD were obtained, respectively. The *K*_a_ between GA and Cu^2+^ (5.724 × 10^2^) as well as GA and NCTD (4.536 × 10^4^) clearly showed that there were strong interactions among GA with Cu^2+^ and NCTD. Both the Δ*G* of GA interacted with Cu^2+^ (-15.7562 kJ/mol) and GA interacted with NCTD (-26.568 kJ/mol) were negative value, which proved that their combination was a spontaneous chemical reaction to form hydrogen bond and so on, rather than a simple physical mixture [46].

To elucidate the self-assembly process of NCTD Gel, a series of spectroscopy studies were performed. The ultraviolet–visible (UV–vis) of GA monomer had a characteristic absorption peak at 256 nm, while NCTD had no absorption peak in the range of 200–400 nm. The characteristic absorption peaks of GA-Cu and NCTD Gel were not significantly shifted compared with GA, indicating that their binding sites were not conjugated to the olefinic bonds of the parent nucleus of GA (Fig. 2C). Thereafter, fourier transform infrared (FT-IR) data showed that GA exhibited typical stretching vibration of -OH group at 3233.86 cm^−1^, C=O group at 1722.07 cm^−1^, COO^−^ group at 1599.11 cm^−1^ and 1422.47 cm^−1^, and the peak at 1036.83 cm^−1^ corresponded to C-O group in the glucuronic acid of GA, respectively (Fig. 2D). After adding Cu^2+^, -OH group exhibited blue shift to 3201.61 cm^−1^; on the other hand, the shape and strength of COO^−^ group changed obviously, indicating that the carboxyl group of GA was likely to be the binding site of coordination bond with Cu^2+^. Then we found that the free NCTD had two typical peaks of C=O group due to the vibrational coupling, and there was only one residue at 1724.63 cm^−1^ after forming Gel (Fig. S2). This change indicated that the dianhydride was hydrolyzed into dicarboxylic acid during the self-assembly of NCTD with GA and Cu^2+^, which was consistent with the active form of NCTD *in vivo* [47]. Compared with the C-O group of GA-Cu at 1032.37 cm^−1^, the C-O group of NCTD Gel decreased obviously at 1007.55 cm^−1^; and the absorption peak of C=O group was evidently enhanced, indicating that NCTD and GA-Cu were assembled successfully and might form hydrogen bond with the hydroxyl group of GA's glucuronic acid. Then the result of circular dichroism (CD) spectra suggested that compared with GA, GA-Cu had an obviously negative Cotton effect, which changed the optical activity of GA's glucuronic acid (Fig. 2E). Combined with FT-IR results, the carboxyl group of GA's glucuronic acid was the binding site of Cu^2+^.

In addition, we performed ^1^H-NMR spectrum to further verify the formation of intermolecular hydrogen bonds and binding sites between GA and NCTD. As revealed in Fig. 2F, it showed a significant change in the shape of the GA's glucuronic acid proton peaks after binding with NCTD, and the proton peak of NCTD carboxyl group disappeared, which was consistent with FT-IR results. It was speculated that the carboxyl group of NCTD was bonded with the hydroxyl group of GA's glucuronic acid in the form of hydrogen bond.

**Fig. 3:**
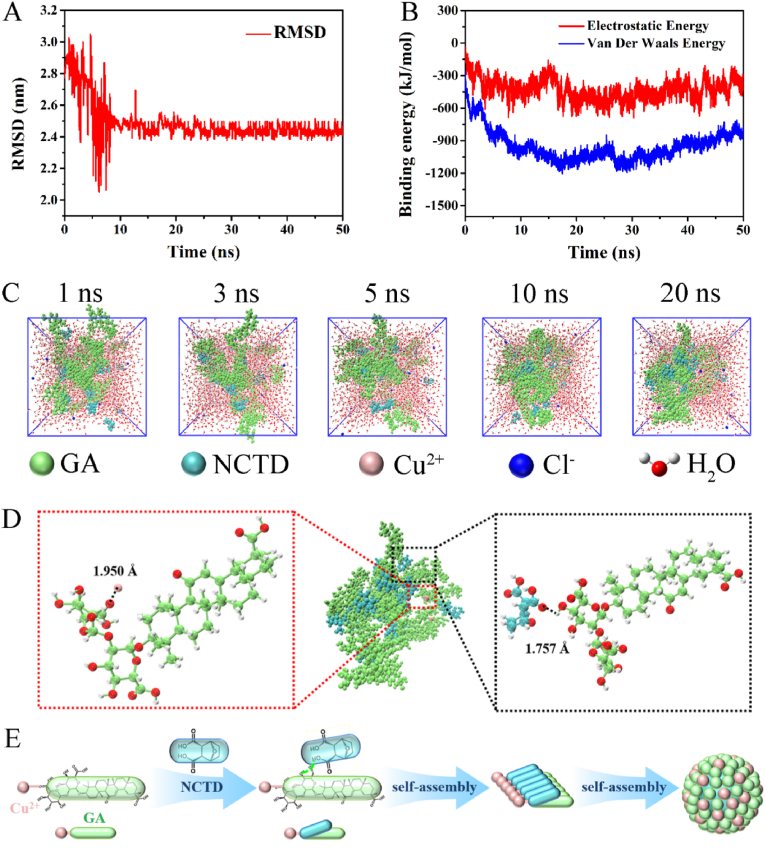
MD simulation of NCTD Gel. **A** Time-dependent changes of RMSD in self-assembly process. **B** The electrostatic energy and van der Waals energy in self-assembly process. **C** The structure changes of NCTD Gel at 1, 3, 5, 10 and 20 ns. **D** The self-assembled structure and intermolecular interaction of NCTD Gel under a stable state (20 ns). **E** The graphic mechanism of self-assembly formation of NCTD Gel.

Furthermore, we used molecular dynamics (MD) simulation to reveal the self-assembly mechanism of NCTD Gel. GA, Cu^2+^ and NCTD were put into the simulation box at a ratio of 2:1:2, and then added water molecules, the number of molecules were shown in Table S3. As presented in Fig. 3A, the root-mean-square deviation (RMSD) result showed that all molecules tended to be stable after 20 ns. Moreover, the solvent accessible surface area (SASA) value was an important factor affecting the stability and compactness of molecular system [48]. We could infer that GA, Cu^2+^ and NCTD formed a stable molecular aggregation from the result of SASA (Fig. S3). Notably, binding energy analysis indicated that electrostatic force and van der Waals force were the main driving forces of self-assembly during the whole process between GA and NCTD (Fig. 3B). The dynamic self-assembling process was showed in Fig. 3C. Thereafter, we analyzed intermolecular interactions of molecular aggregations at 20 ns. As depicted in Fig. 3D, Cu^2+^ formed a coordination bond with carboxyl group of GA's second glucuronic acid; NCTD's carboxyl group formed a hydrogen bond with hydroxyl group of GA's first glucuronic acid, respectively. MD simulation clearly displayed the ternary self-assembly process and mechanism, which was accordance with the above experimental results.

Combining the above ITC, spectroscopy studies, NMR results with MD simulation calculation, we proposed a possible self-assembly process of NCTD Gel as shown in Fig. 3E. The ternary carrier-free hydrogel was formed by various noncovalent interactions including the coordination interactions between GA and Cu^2+^, the intermolecular hydrogen bonds between GA and NCTD, respectively. Subsequently, trimers were further assembled into higher-order aggregates, which crosslinked to form the uniform spherical particles and presented as an injectable hydrogel with excellent rheological properties (Fig. 1G-L).

### 3.3 In vitro tumor-microenvironment modulation capacity and antitumor efficacy

**Fig. 4:**
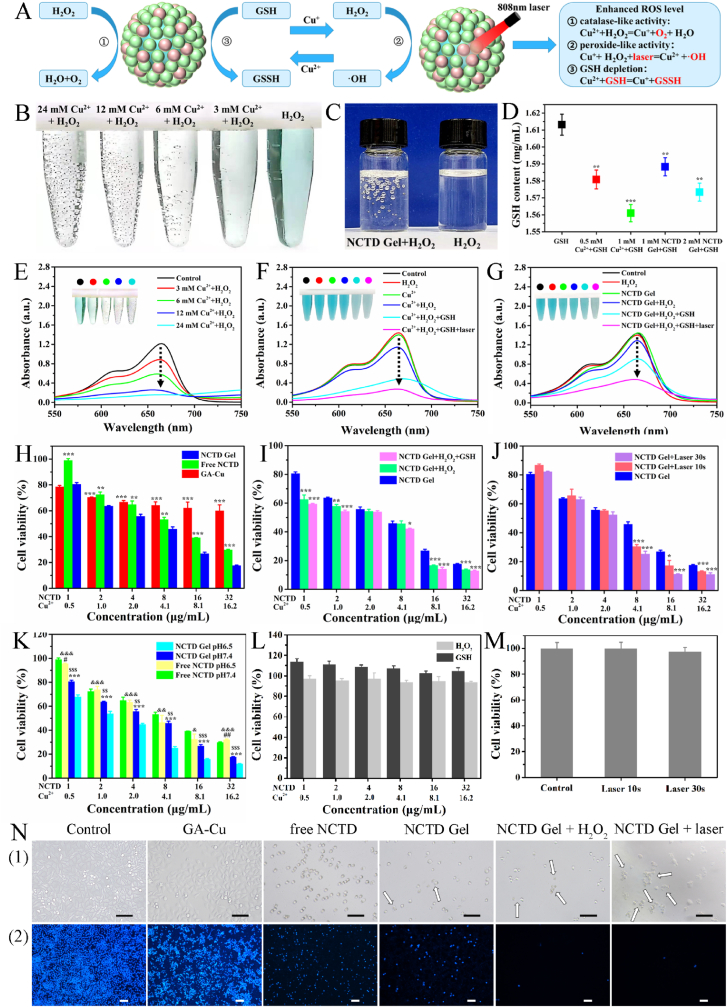
Tumor-microenvironment modulation capacity and cytotoxicity of NCTD Gel. **A** Schematic illustration of the photo-Fenton-like activity of NCTD Gel. **B** Photographs of different concentrations of Cu^2+^ mixed with H_2_O_2_ and MB. **C** Photographs of NCTD Gel mixed with H_2_O_2_, pure H_2_O_2_ was as control. **D** Content of GSH mixed with different concentrations of Cu^2+^ and NCTD Gel, the reaction time was 30 min **P < 0.01, ***P < 0.001 compared with GSH. **E** UV–vis changes of MB mixed with H_2_O_2_ and different concentrations of Cu^2+^, the reaction time was 30 min. **F** Cu^2+^ (1 mM) and **G** NCTD Gel (2 mM) mixed with MB underwent different treatment, UV–vis changes of MB after 30 min. **H** Cell viability of HepG2 cells treated with a series concentration of NCTD Gel, Free NCTD and GA-Cu for 72 h **P < 0.01, ***P < 0.001 compared with NCTD Gel. **I** Cell viability of HepG2 cells treated with a series concentration of NCTD Gel mixed with H_2_O_2_ and GSH for 72 h *P < 0.05, **P < 0.01, ***P < 0.001 compared with NCTD Gel. **J** Cell viability of HepG2 cells treated with a series concentration of NCTD Gel + laser at different time (1.0 W/cm^2^) for 72 h *P < 0.05, ***P < 0.001 compared with NCTD Gel. **K** Cell viability of HepG2 cells treated with a series concentration of NCTD Gel and Free NCTD at PH 7.4 or 6.5 for 72 h ***P < 0.001 compared with NCTD Gel pH6.5, ^#^*P* < 0.05, ^##^*P* < 0.01 compared with free NCTD pH7.4, ^&^*P* < 0.05, ^&&^*P* < 0.01, ^&&&^*P* < 0.001 compared with NCTD Gel pH6.5, ^$$^*P* < 0.01, ^$$$^*P* < 0.001 compared with free NCTD pH7.4. **L** Cell viability of HepG2 cells treated with a series concentration of H_2_O_2_ or GSH for 72 h. **M** Cell viability of HepG2 cells treated with laser at different time (1.0 W/cm^2^) for 72 h. **N** (1) Optical observation of HepG2 cells with different treatments. **N** (2) DAPI staining of HepG2 cells with different treatments. Scale bar = 50 μm. All data were presented as the mean ± SD, (n = 3).

The Fenton-like activity of NCTD Gel was predicted in Fig. 4A. At first, NCTD Gel could degrade the H_2_O_2_ to Cu^+^ and O_2_ (catalase-like activity). Meanwhile, O_2_ could fight against the hypoxia in TME. On the other hand, Cu^+^ could further degrade H_2_O_2_ and generate ∙OH (peroxide-like activity) to kill the tumor cells, and the cytotoxic effect were enhanced under laser irradiation. In addition, NCTD Gel could deplete GSH to protect the generated ROS [28].

We observed the TME modulation ability of Cu^2+^ and NCTD Gel. As shown in Fig. 4B and E, when different concentrations of Cu^2+^ mixed with H_2_O_2_ and methylene blue (MB), the rapid production of O_2_ could be seen with the increase of Cu^2+^ concentration, and the color turned lighter gradually, the absorption peak of MB decreased gradually at the same time. As the free radical ·OH was a typical product of Fenton-like reaction and MB was an indicator for the detection of ·OH, it could be proved that Cu^2+^ had catalase-like activity to enhance the production of O_2_ and ·OH. Furthermore, there were no obvious variations in the absorption peak of MB when it was co-incubated with water, H_2_O_2_ and Cu^2+^; but the absorption peak rapidly decreased along with H_2_O_2_ and GSH were successively added into Cu^2+^; especially, the absorption peak was the lowest when 808 nm laser was added (Fig. 4F). It was indicated that the addition of GSH and laser could greatly improve the reaction's efficiency. Thereafter, the content of GSH was detected by an indicator DTNB (5,5′-Dithiobis-(2-nitrobenzoic acid)). As Fig. 4D presented, when Cu^2+^ mixed with GSH, an increased GSH depletion was observed with the increased Cu^2+^ concentration. It confirmed that Cu^2+^ could deplete GSH to protect the ROS produced.

More importantly, the similar phenomenon happened after Cu^2+^ was replaced by NCTD Gel. Along with NCTD Gel mixed with H_2_O_2_, an obvious O_2_ bubbles could be observed, whereas pure H_2_O_2_ solution had no such phenomenon (Fig. 4C). Moreover, under the same conditions, NCTD Gel produced less ·OH (Fig. 4G) and depleted less GSH than Cu^2+^ (Fig. 4D), indicating that NCTD Gel had slowly controlled release character compared with Cu^2+^. Remarkably, more ·OH was produced after the laser irradiation (Fig. 4G), indicating that NCTD Gel had a good photo-catalyst character with promoting Fenton-like reaction activity under laser irradiation. In a word, those results exhibited that both Cu^2+^ and NCTD Gel had good laser-mediated photo-Fenton-like reaction to regulate TME; moreover, NCTD Gel had the advantage of excellent photosensitive property, slow controlled and longtime release effect as an injectable hydrogel with excellent mechanical characters, so it could be beneficially applied in CDT for tumor treatment.

Encouraged by the exciting TME modulation capacity, the cytotoxicity of NCTD Gel, free NCTD and GA-Cu was evaluated in HepG2 cells, respectively (Fig. 4H). Both NCTD Gel and free NCTD inhibited cell proliferation in a dose-dependent manner. In addition, NCTD Gel exhibited a more significant cytotoxicity effect than free NCTD. Among them, GA-Cu showed weak cytotoxicity at the tested concentration.

Thereafter, cytotoxicity assay of NCTD Gel was observed in response to H_2_O_2_ and GSH stimuli, laser irradiation, and pH changes, respectively. When H_2_O_2_ and GSH of the same concentration as Cu^2+^ were added into NCTD Gel, the cytotoxicity was significantly enhanced; in contrast, the group without GSH showed relatively weak cytotoxicity, and single H_2_O_2_ and GSH had little cytotoxic effect (Fig. 4I and L). The results showed that synergetic chemical reaction between NCTD Gel and H_2_O_2_/GSH improved its cytotoxicity. For photocytotoxicity (Fig. 4J and M), under different laser irradiation time, the cell viability was gradually reduced with the change of NCTD Gel concentrations (8, 16, 32 μg mL^−1^ of NCTD). With the longer the irradiation time, the stronger the cytotoxicity of NCTD Gel was exhibited; whereas the single laser irradiation did not influence the cell viability, which further confirmed the result that NCTD Gel presented synergetic chemo-photo therapeutic effect. Meanwhile, the cytotoxic effect of NCTD Gel was detected in media with a pH value of 7.4 or 6.5. As shown in Fig. 4K, the cell viability of NCTD Gel at pH 6.5 was much lower than pH 7.4. However, there was almost no difference of free NCTD between the two kinds of pH, indicating that Cu^2+^ in NCTD Gel was beneficial to exert Fenton-like reaction and to kill tumor cells in weakly acidic TME. The results were consistent with the study that weakly acidic environment was beneficial for Cu^2+^ to produce Fenton-like reaction [49]. Furthermore, we performed the release experiments of NCTD Gel in normal and tumor microenvironments. We tested the content of organic component GA released from the NCTD Gel. As presented in Fig. S4, the release of NCTD Gel in TME was larger than that in the normal microenvironment. After the addition of 808 nm laser irradiation, the release of NCTD Gel in TME was faster. The results showed that on the one hand, weakly acidic of TME and 808 nm laser irradiation could facilitate the release of pharmacodynamic components in NCTD Gel, on the other hand, it could also improve the efficiency of Cu^2+^’s photo-Fenton-like reaction, which worked together to make NCTD Gel play a role and was consistent with the results of pharmacodynamic experiment *in vitro*. Moreover, the cellular morphology of HepG2 cell was influenced visually after incubated with NCTD Gel under different treatments (Fig. 4N (1)). The broken cytomembrane and morphology shrink were induced by NCTD Gel; whereas free NCTD only induced morphology shrink. Meanwhile, when laser was added in NCTD Gel, the cytomembrane was significantly broke. Furthermore, the results of 4′,6-Diamidino-2-phenylindole (DAPI) staining were consistent with MTT assay (Fig. 4 N (2)). In general, all results indicated that NCTD Gel had excellent TME modulation capacity and antitumor effect *in vitro*.

### 3.4 In vitro cell death mechanism analysis of NCTD Gel

**Fig. 5:**
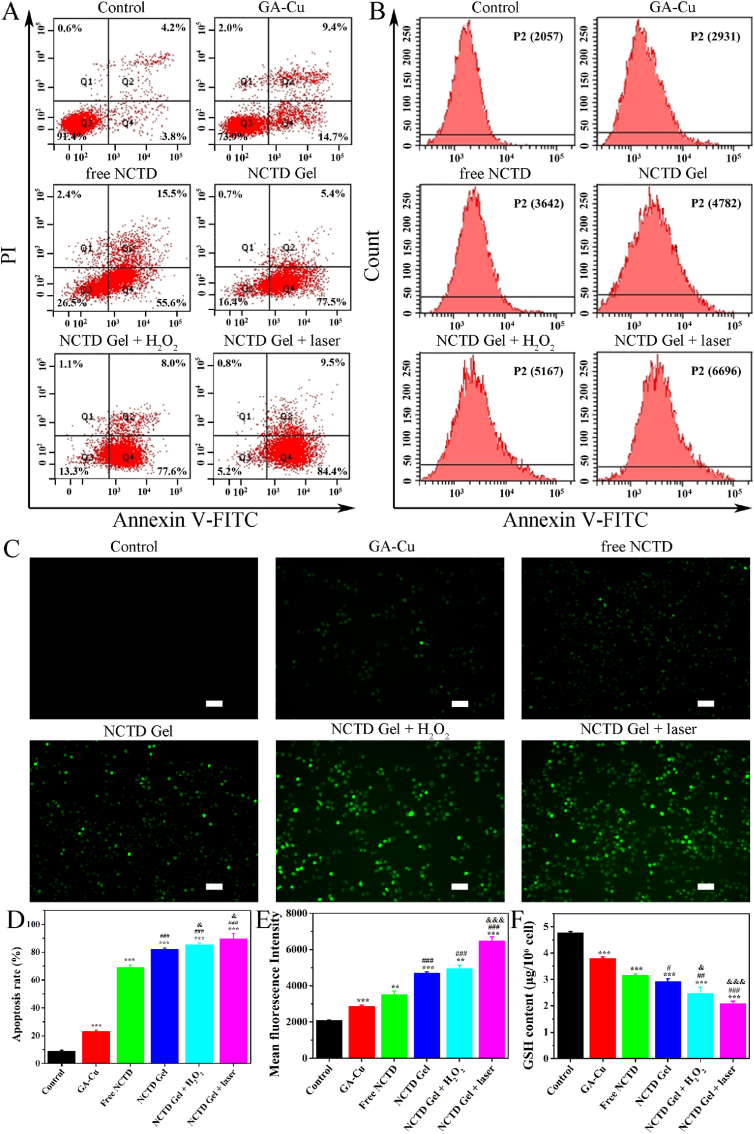
*In vitro* cell death mechanism analysis of NCTD Gel. **A** and **D** Flow cytometric analysis of HepG2 cells apoptosis induced by different treatments with Annexin V-FITC/PI staining. **B** and **E** Flow cytometric analysis of HepG2 cells ROS induced by different treatments with DCFH-DA staining. **C** DCFH-DA staining of HepG2 cells with different treatments. Scale bar = 50 μm. **F** Intracellular GSH level of HepG2 cells with different treatments measured by GSH Assay Kit. All data were presented as the mean ± SD (n = 3), *P < 0.05, **P < 0.01, ***P < 0.001 compared with control, ^#^*P* < 0.05, ^##^*P* < 0.01, ^###^*P* < 0.001 compared with free NCTD, ^&^*P* < 0.05, ^&&^*P* < 0.01, ^&&&^*P* < 0.001 compared with NCTD Gel.

Inspired by NCTD Gel-induced cell death *in vitro*, we attempted to reveal its mechanism of antitumor efficacy. Thereafter, we used flow cytometric analysis to confirm HepG2 cells apoptosis induced by the current treatments. As depicted in Fig. 5A and D, GA-Cu, free NCTD and NCTD Gel treatment induced cells apoptosis to about 23%, 69% and 82%, respectively. On the other hand, following the H_2_O_2_ and laser were added into NCTD Gel, the apoptosis response was elevated to 86% and 90%, respectively. The above phenomenon indicated that high toxic ·OH of NCTD Gel accelerated cell apoptosis under laser irritation and further demonstrated its TME modulation capacity *in vitro*. Additionally, as shown in Fig. 5B and E, flow cytometric analysis was used to detect the content of intracellular ROS under different treatments. The content of ROS in NCTD Gel + laser was the highest compared with other treatment groups. To further verify the production of ROS, we also used DCFH-DA to observe the generation of intracellular ROS after incubation with GA-Cu, free NCTD and NCTD Gel for 12h, respectively (Fig. 5C). Cells treated with NCTD Gel showed a brighter green fluorescence than GA-Cu and free NCTD. In addition, following the addition of H_2_O_2_ and laser, the green fluorescence was obviously enhanced. The results were consistent with flow cytometric analysis. Furthermore, intracellular evolved O_2_ was detected by an O_2_ probe (RDPP). As shown in Fig. S5, compared with PBS and free NCTD group, cells treated with GA-Cu and NCTD Gel presented quenched red fluorescence, which indicated the hypoxia-resistance capacity of NCTD Gel, especially under H_2_O_2_ and laser induced conditions. Moreover, we detected the content of intracellular GSH using DTNB probe. As shown in Fig. 5F, GSH in NCTD Gel + laser was the lowest (2.08 ± 0.09 μg/10^6^ cell), and NCTD Gel with H_2_O_2_ was the following. GSH content was opposite to the trend of intracellular ROS content. The above results confirmed that NCTD Gel did have Fenton-like activity, which could promote cells to produce ROS and deplete excess intracellular GSH. NCTD Gel had promising property to regulate TME and apply as CDT for tumor.

### 3.5 In vivo antitumor efficacy

**Fig. 6:**
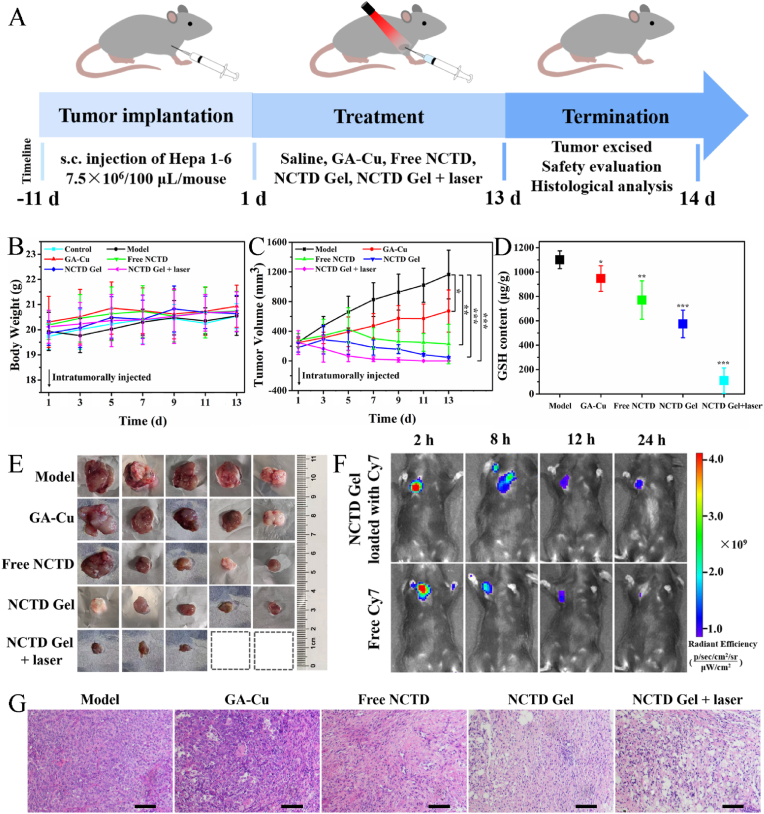
*In vivo* antitumor activity. **A** Schematic illustration of the experimental design to evaluate the therapeutic effect (n = 5). **B** Time-dependent changes of body weight. **C** Curves of tumor growth volume during the treated process. **D** GSH content of tumor tissues measured by GSH Assay Kit. **E** Images of tumor tissues after various treatments for 13 days. **F** Mice imaged *in vivo* with fluorescence at various times after receiving an intratumor injection of NCTD Gel. **G** Histological analysis of H&E of tumor tissues. Scale bars: 50 μm. All data were presented as the mean ± SD, *P < 0.05, **P < 0.01, ***P < 0.001 (n = 5).

Subsequently, encouraged by the promising *in vitro* TME modulation capacity and antitumor effect of NCTD Gel, we further evaluated its therapeutic efficacy *in vivo* on Hepa 1-6 tumor-bearing mice via intratumoral injection. The experimental process was shown in Fig. 6A. The body weight and tumor volume were recorded to evaluate the capacity and the safety during the treated process. After various treatments, the mice exhibited similar normal body weight with little weight loss (Fig. 6B), implying that all samples had no obvious toxic. And different degrees of tumor inhibition were detected in all drug treatment groups (Fig. 6C). The NCTD Gel + laser group showed the best effect on the inhibition of tumor growth, followed by NCTD Gel group which was better than that of free NCTD group. Among them, GA-Cu group showed weak inhibition of tumor growth. Meanwhile, we detected the GSH content of tumor tissues using DTNB probe. As depicted in Fig. 6D, GSH in NCTD Gel + laser group was the lowest (109.16 ± 104.14 μg/g), and NCTD Gel was followed; in contrast, model group was the highest (1101.09 ± 73.04 μg/g). GSH content was consistent with the antitumor effect of each sample, which was the same trend as the tumor volume in all groups. In addition, the results of intratumoral GSH content was accordance with the intracellular test results, demonstrating the Fenton-like reaction of NCTD Gel occurred on tumor cells *in vitro* and tissues *in vivo*. Notably, images of tumor tissues after 13 days' treatment also confirmed the different antitumor activity of each group (Fig. 6E). Especially, the tumor tissues were obviously reduced and even disappeared with no recurrence in NCTD Gel + laser group, which displayed the promising anti-tumor effect. We further performed tumor histological analysis of each group, and the results were shown in Fig. 6G. By comparing the H&E staining of different treatment, it could be found that both NCTD Gel + laser and NCTD Gel groups had the better severe damage to tumor tissues, especially NCTD Gel + laser group exhibited the typical necrotic features and the apparent vacuolation of tumor cells. The tumors treated with free NCTD exhibited a higher proportion of necrotic cells than those treated with GA-Cu, whereas the saline control exhibited no obvious necrosis. Furthermore, as presented in Fig. 6F, the fluorescence of NCTD Gel loaded with Cy7 was significantly stronger in the tumor compared to free Cy7 at various times after receiving an intratumor injection. It was suggested that NCTD Gel with perfect viscoelastic property and stability could maintain a long-time retention in tumor and display antitumor efficacy by regulating TME and inducing apoptosis. Taken together, evidence from tumor volume, GSH content, image of tumor tissue and H&E staining totally affirmed that NCTD Gel + laser and NCTD Gel groups had the superior antitumor effect in comparison to free NCTD on Hepa 1-6 tumor-bearing mice model. It is suggested that the NCTD Gel's retention character and laser-mediated photo-Fenton-like reaction to regulate TME are beneficial for therapeutic efficacy.

### 3.6 Antitumor mechanism analysis of NCTD Gel

Based on the excellent antitumor efficacy of the NCTD Gel *in vitro* and *in vivo*, we further analyzed its multi-pathway mechanism from three aspects: apoptosis, cuproptosis and anti-inflammation. For example, NCTD was a clinical antitumor drug which could induce cell apoptosis [40]; and Cu^2+^ had Fenton-like reaction which could further promote cell apoptosis [28]. Especially, recent studies indicated that Cu^2+^ could kill tumor cells through cuproptosis, which was a novel mechanism of cell death that was distinct from ferroptosis [31]. On the other hand, a large number of inflammatory cells and cytokines (such as TNF-α, IL-1β and IL-6) existed in TME, which played an important role in the malignant cells’ proliferation, invasion and metastasis [50]. To solve the inflammation challenge, GA displayed well anti-inflammation and regulation of TME effects, which were helpful in inhibiting tumor invasion and metastasis.

**Fig. 7:**
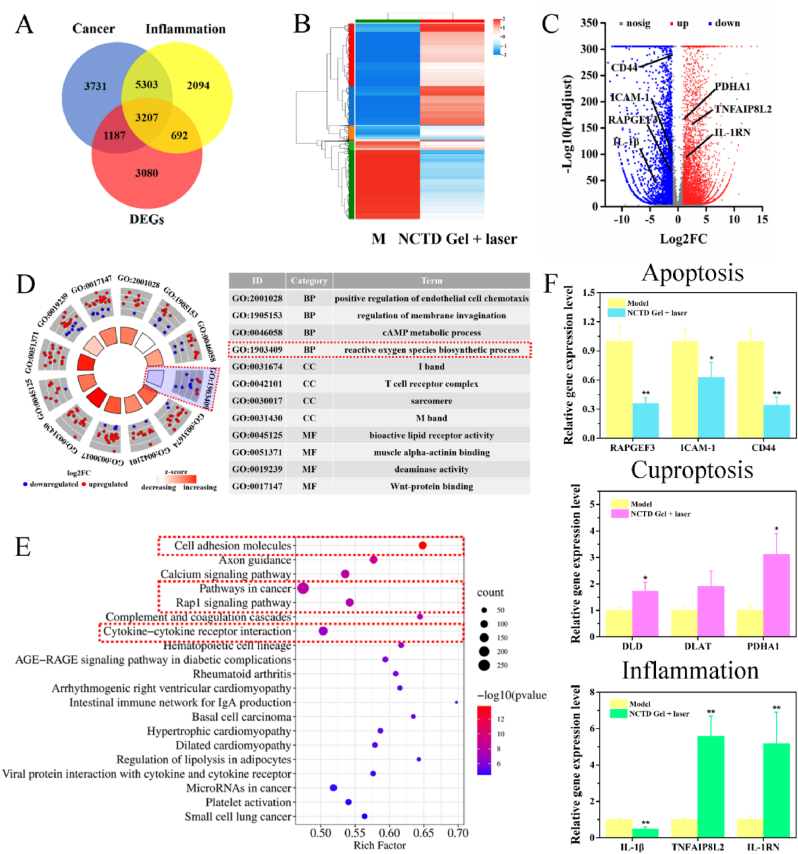
RNA-seq test and qPCR analysis. **A** Venn analysis of DEGs, Cancer and Inflammation. **B** Hierarchical clustering analysis of DEGs. **C** Volcano plot of DEGs. Blue and red represented down-regulated and up-regulated genes, respectively. **D** GO functional analysis of DEGs. Blue and red represented down-regulated and up-regulated genes in each pathway, respectively. **E** KEGG enrichment analysis of DEGs. The bubble size reflected the gene counts enriched in each term, and color represented the *p* value. **F** The relative gene expression levels of RAPGEF3, ICAM-1, CD44, DLD, DLAT, PDHA1, IL-1β, TNFAIP8L2, IL-1RN of tumors in model and NCTD Gel + laser groups. Data were presented as the mean ± SD, *P < 0.05, **P < 0.01, ***P < 0.001 (n = 3).

To clearly reveal the above multi-pathway mechanism, we performed tumors' RNA-seq analysis on model and NCTD Gel + laser groups to screen the differentially expressed genes (DEGs). As shown in Fig. 7A, blue circle related to 13428 cancer genes, yellow circle represented 11296 inflammation genes from GeneCards database, respectively. Thereafter, red circle represented a total of 8166 DEGs of model and NCTD Gel + laser groups, including 5094 up-regulated genes and 3072 down-regulated genes (Fig. S6). And the 8166 DEGs were intersected with cancer and inflammation genes, resulting in cancer related to 4394 and inflammation related to 3899 overlapping genes, respectively. Among them, 3207 DEGs were associated with both cancer and inflammation. Results showed that inflammation and cancer were closely linked, and NCTD Gel had the potential to treat cancer and inflammation simultaneously. As depicted in Fig. 7B and C, hierarchical clustering analysis and volcano plot of DEGs indicated that there were significant differences in gene expression levels between model and NCTD Gel + laser groups. Compared with model group, the Gel + laser treated group's tumor apoptosis and metastasis related genes CD44, ICAM-1 and RAPGEF3 were not only down-regulated, but also their pro-inflammation related gene IL-1β was down-regulated. Meanwhile, both anti-inflammatory related genes TNFAIP8L2, IL-1RN and cuproptosis related gene PDHA1 were up-regulated in the NCTD Gel + laser group. Additionally, Kyoto Encyclopedia of Genes and Genomes (KEGG) analysis (Fig. 7D) and Gene Ontology (GO) analysis (Fig. 7E) were conducted on DEGs to further determine the effect of genes on pathways. The results further demonstrated that NCTD Gel synergy functioned primarily by regulating tumor- and inflammation-related pathways. For example, reactive oxygen species biosynthetic process, Rap1 signaling pathway, Cell adhesion molecules and Cytokine-cytokine receptor interaction were enriched, respectively. The results were consistent with the effects of NCTD Gel on inducing cell apoptosis, cuproptosis and anti-inflammation. Furthermore, we detected the expression levels of some vital genes to verify the reliability of RNA-seq analysis by qPCR, including RAPGEF3, ICAM-1, CD44, DLD, DLAT, PDHA1, IL-1β, TNFAIP8L2, IL-1RN (Fig. 7F). Among them, RAPGEF3, ICAM-1, CD44 and IL-1β were down-regulated; DLD, DLAT, PDHA1, TNFAIP8L2 and IL-1RN were up-regulated after treatment, respectively. The qPCR results were consistent with RNA-seq experiments.

**Fig. 8:**
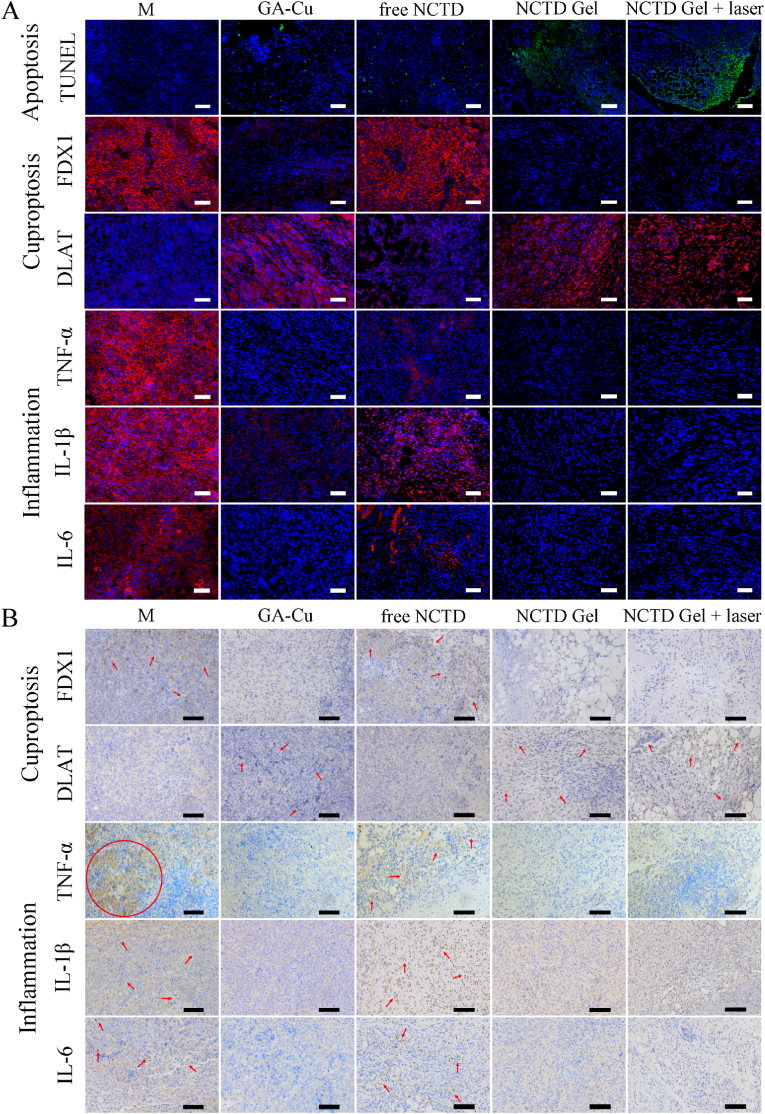
Immunofluorescence and immunohistochemical analysis. **A** TUNEL, FDX1, DLAT, TNF-α, IL-1β, IL-6 staining of tumors after various treatments. Scale bar, 50 μm. **B** Immunohistochemical analysis of FDX1, DLAT, TNF-α, IL-1β, IL-6 of tumors after various treatments. Scale bar, 50 μm.

Encouraged by the RNA-seq and qPCR results, both immunofluorescence and immunohistochemistry were used to further verify the synergistically antitumor mechanism of NCTD Gel. As presented in Fig. 8A, we used TdT-mediated dUTP nick end labeling (TUNEL) staining to analyze tumor apoptosis. Blue fluorescence represented normal tumor cells, while green fluorescence was produced by reaction of nucleus and TUNEL when cells underwent apoptosis. TUNEL staining indicated that tumor tissues of NCTD Gel/NCTD Gel + laser groups showed broadly green fluorescence, which indicated that more cell apoptosis was occurred than free NCTD and GA-Cu groups. And model group displayed almost totally blue fluorescence and had no obvious tumor cell apoptosis. The superior antitumor efficacy and induction of cell apoptosis of NCTD Gel + laser was also supported by TUNEL staining, which was consistent with the RNA-seq, qPCR and flow cytometry results. In addition, red fluorescence represented the positive expression of immunofluorescence of FDX1, DLAT, TNF-α, IL-1β and IL-6. Among them, FDX1 and DLAT were iron-sulfur cluster protein and lipoylated protein in tumor cells, respectively. The loss of FDX1 and aggregation of DLAT were observed in GA-Cu, NCTD Gel and NCTD Gel + laser groups, which were the representative characteristic of cuproptosis. And it was directly proved that NCTD Gel could treat tumor through cuproptosis. TNF-α, IL-1β and IL-6 were proinflammatory factors and their red fluorescence in GA-Cu, NCTD Gel and NCTD Gel + laser treated groups was all much weaker than model and free NCTD groups. In general, NCTD Gel + laser displayed the best medication effect on apoptosis, cuproptosis and anti-inflammation.

Thereafter, immunohistochemical analysis was further carried out to prove the results of immunofluorescence (Fig. 8B). Proteins related to cuproptosis and inflammation were displayed positive expression and turned brown. Therefore, the brown distribution in tumor tissues could be used to compare the antitumor and anti-inflammatory effect of each group. In GA-Cu, NCTD Gel and NCTD Gel + laser treated groups, FDX1 showed almost no positive brown expression; while DLAT had more brown distribution than model and free NCTD groups, which was embodied the characteristics of cuproptosis. Moreover, as for inflammatory factors TNF-α, IL-1β and IL-6, the brown stain of model group showed a wider distribution and deeper coloring than others, indicating severe inflammatory cell infiltration of TME. Among the treated groups, the positive brown expression in the free NCTD group was similar to the model group. It was suggested that free NCTD had a certain antitumor effect, but it could not reduce the inflammation in TME. In contrast, there was almost no positive brown expression in tumors of GA-Cu, NCTD Gel and NCTD Gel + laser groups. It was further proved that GA of NCTD Gel did display well anti-inflammatory effect and could downregulate the level of TNF-α, IL-1β and IL-6, which was accordance with the previous GA anti-inflammatory study [33]. The results were consistent with immunofluorescence, which also indicated that NCTD gel had good anti-tumor and anti-inflammatory effects. These findings demonstrated that ternary self-assembled NCTD Gel had the triple effects of promoting tumor cell apoptosis, cuproptosis and anti-inflammation, which might simultaneously contribute to play a vital role in inhibiting tumor proliferation, invasion and metastasis.

### 3.7 Biosafety evaluation *in vitro* and *in vivo*

**Fig. 9:**
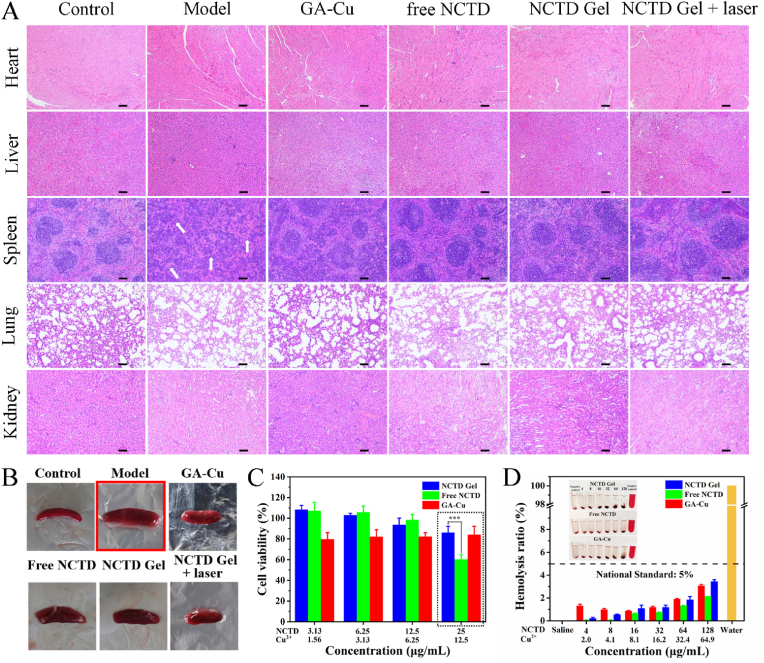
Biosafety evaluation *in vitro* and *in vivo*. **A** The images of H&E staining of major organs after various treatments for 13 days. The scale bar = 50 μm. **B** Morphological change of spleen after various treatments for 13 days. **C** Cell viability (72 h) of MDCK cells incubated with different concentrations of NCTD Gel, free NCTD and GA-Cu. **D** Hemolysis assay of RBCs treated with deionized water, PBS and different concentrations of NCTD Gel, free NCTD and GA-Cu for 1 h (Inset: Photographs after centrifugation). All data were presented as the mean ± SD, *P < 0.05, **P < 0.01, ***P < 0.001 (n = 3).

The biosafety of drugs was a key factor affecting its clinical application. Based on the excellent antitumor effect of the NCTD Gel *in vitro* and *in vivo*, we further evaluated its safety through H&E staining of different organs, cytotoxicity and hemolysis assay. As presented in Fig. 9A and S7, compared with control group, no obvious pathological changes were found in the heart, liver, spleen, lung and kidney harvested after 13 days of treatment in each administration group. In contrast, spleen tissue of model group displayed splenomegaly in the appearance (Fig. 9B). Meanwhile, the result of model groups’ spleen H&E staining showed indistinct boundaries between red and white pulps, white pulps dissociation, and disorderly arrangement of cells, which was obviously different than normal control and other treated groups. As the spleen was the largest peripheral immune organ, the changes of model group suggesting tumor-induced immune dysfunction, possibly accompanied by an inflammatory response in the spleen [51,52]. Herein, spleen tissue remained intactly in administration groups, which reflected that NCTD Gel could effectively anti-tumor and anti-inflammation to block the spleen enlargement induced by cancer. Furthermore, the *in vitro* cytotoxicity of NCTD Gel, free NCTD and GA-Cu was detected by MTT assay using kidney MDCK cells (Fig. 9C). After 72 h of incubation, the inhibition rate of GA-Cu and NCTD Gel on MDCK cells was always less than 20%, indicating that they had good biosafety. While the inhibition rate of free NCTD was 40% at the concentration of 25 μg/mL, proving that the self-assembly of GA-Cu and free NCTD into a hydrogel could improve the biosafety. In our previous experiments, we found that NCTD Gel had stronger cytotoxicity than free NCTD on HepG2 cells, indicating that GA-Cu could synergize with free NCTD to play an anti-tumor effect which was accordance with the previous report that copper could induce cell death [31]. While on MDCK cells, NCTD Gel attenuated the cytotoxicity of free NCTD to a certain extent. This might be due to the TME was weak acidity, H_2_O_2_ overexpression and hypoxia, which was beneficial for Cu^2+^ to produce Fenton-like reaction. However, normal tissues and cells such as MDCK cells did not possess these characteristics. As depicted in Fig. 9D, hemolysis assay showed that even the concentration was as high as 128 μg/mL, there was no obviously hemolytic activity on rat erythrocytes in all administration groups. The hemolysis rate of NCTD Gel was much lower than the internationally recognized standard (5%), indicating its good biocompatibility. Due to the constituent materials of NCTD Gel were originated from the natural small molecule and clinical agents, NCTD Gel not only possessed significant antitumor effect, but also had well biocompatibility. It did not involve the carrier risk and was green preparation, which was suitable for the future clinical transformation.

## 4 Conclusion

We successfully developed a metal ions-mediated natural small molecules carrier-free injectable NCTD Gel, which were formed by co-assembly of GA, Cu^2+^ and NCTD to achieve CDT of cancer. In this ternary hydrogel system, the three components played synergistic and indispensable roles in both hydrogel formation and pharmacodynamics. Briefly, GA, Cu^2+^ and NCTD were co-assembled through coordination bonds and hydrogen bonds to form NCTD Gel. It had perfect viscoelastic property and stability, which could maintain a long-time retention in tumor, thereby enhancing the antitumor efficacy. Meanwhile, GA exerted anti-inflammatory effect to modulate TME, NCTD and Cu^2+^ induced apoptosis, and Cu^2+^ could simultaneously kill tumor cells through cuproptosis. In short, ternary self-assembled NCTD Gel not only comprehensively utilized the characteristics of TME, but also modulated TME, and finally achieved multi-channel inhibition of tumor proliferation, invasion and metastasis through synergistic apoptosis/cuproptosis and anti-inflammation. Although, the high-end nano-drugs and gene therapy for tumor treatment have achieved great progress, the complex preparation and high-cost treatments currently hinder the worldwide clinical use. NCTD Gel not only have good biocompatibility, but also its preparation process is green, easy, low cost and without modification, which exhibit great potential for clinical transformation in cancer treatment.

## Data availability

Data will be made available on request.

## Ethics approval and consent to participate

Animal experiments conform to the guidelines for the Care and Use of Experiment Animals in Institutional Animal Care and Use Committee (IACUC) at the Beijing University of Chinese Medicine, China (protocol number BUCM-2019090701-3031).

## CRediT authorship contribution statement

**Wenmin Pi:** Methodology, Writing – original draft, Writing – review & editing. **Linying Wu:** Conceptualization, Validation, Formal analysis. **Jihui Lu:** Methodology, Investigation, Resources, Data curation. **Xiaoyu Lin:** Methodology, Writing – review & editing. **Xuemei Huang:** Methodology, Writing – review & editing. **Zhijia Wang:** Investigation, Formal analysis. **Zhihua Yuan:** Visualization, Resources. **Hailing Qiu:** Formal analysis, Resources. **Jianglan Zhang:** Methodology, Supervision. **Haimin Lei:** Supervision, Resources. **Penglong Wang:** Methodology, Writing – review & editing, Project administration, Funding acquisition.

## Declaration of competing interest

The authors declared that they have no conflicts of interest to this work. We declare that we do not have any commercial or associative interest that represents a conflict of interest in connection with the work submitted.
