# Supplementary material for: A metal ions-mediated natural small molecules carrier-free injectable hydrogel achieving laser-mediated photo-Fenton-like anticancer therapy by synergy apoptosis/cuproptosis/anti-inflammation
Source: Bioact Mater. 2023 Jul 5;29:98–115. doi: 10.1016/j.bioactmat.2023.06.018 (PMC10345197; doi:10.1016/j.bioactmat.2023.06.018)
Supplement: Multimedia component 1 [file mmc1.docx]

Supporting Information

A metal ions-mediated natural small molecules carrier-free injectable hydrogel achieving laser-mediated photo-Fenton-like anticancer therapy by synergy apoptosis/cuproptosis/anti-inflammation

Wenmin Pi^1^, Linying Wu^1^, Jihui Lu^1^, Xiaoyu Lin^1^, Xuemei Huang^1^, Zhijia Wang^1^, Zhihua Yuan^1^, Hailing Qiu^1^, Jianglan Zhang^2^, Haimin Lei^1^, Penglong Wang^1^*

*^1^School of Chinese Pharmacy, Beijing University of Chinese Medicine, Beijing 102488, China*

*^2^School of Traditional Chinese Medicine, Beijing University of Chinese Medicine, Beijing 102488, China*

*Corresponding author. E-mail: wpl581@126.com (P.W.)

**1. Materials and methods**

**Materials.** Glycyrrhizic acid (GA, C_42_H_62_O_16_, MW 822.93, ≥95%) was purchased by Shanghai Yuanye Bio-Technology Co., Ltd (Shanghai, China). Copper chloride (CuCl_2_·2H_2_O, MW 170.48, ≥99%) was purchased by Fuchen (Tianjin) Chemical Reagent Co., Ltd (Tianjin, China). Norcantharidin (NCTD, C_8_H_8_O_4_, MW 168.15, ≥98%) was purchased by Shanghai Macklin Biochemical Co., Ltd (Shanghai, China). Methylene blue (MB) was purchased by Shanghai Macklin Biochemical Co., Ltd (Shanghai, China). 3-(4,5-Dimethyl-thiazol-2-yl)-2,5-diphenyl tetrazolium bromide (MTT) was purchased by Beijing Biorigin Biotechnology Co., Ltd (Beijing, China). Reduced glutathione (GSH) assay kit was purchased by Beijing Solarbio Science & Technology Co., Ltd (Beijing, China). Annexin V-FITC apoptosis detection kit was purchased by Beyotime Biotech Inc (Shanghai, China). Reactive oxygen species (ROS) assay kit was purchased by Beyotime Biotech Inc (Shanghai, China). Sulfo-Cyanine7 (Cy7) was purchased by Dalian Meilunbio Biotechnology Co., Ltd (Dalian, China). 4′,6-Diamidino-2-phenylindole (DAPI) was purchased by Beyotime Biotech Inc (Shanghai, China). TdT-mediated dUTP nick end labeling (TUNEL) was purchased by Wuhan Servicebio Technology Co., Ltd (Wuhan, China). The HepG2, Hepa1-6 and MDCK cells were purchased by the Chinese Academy of Medical Sciences & Peking Union Medical College. Fetal bovine serum (FBS) and Dulbecco’ modified Eagle’ medium (DMEM) was obtained from Biological Industries (Kibbutz Beit Haemek, Israel). C57BL/6J mice (female, 20 g ± 1 g) were purchased by Beijing Vital River Laboratory Animal Technology Co., Ltd (Beijing, China) and conform to the guidelines for the Care and Use of Experiment Animals in Institutional Animal Care and Use Committee (IACUC) at the Beijing University of Chinese Medicine, China (protocol number BUCM-2019090701-3031). Anti-FDX1 antibody was purchased by Wuhan ABclonal Technology Co., Ltd (Wuhan, China). Anti-DLAT antibody was purchased by Beijing Solarbio Science & Technology Co., Ltd (Beijing, China). Anti-TNF-α, Anti-IL-1β and Anti-IL-6 antibodies were purchased by Wuhan Servicebio Technology Co., Ltd (Wuhan, China). All the reagents were not further purified throughout the experiment.

**Rheology test.** The rheological behavior test was experimented by the rheometer (MCR 302, Aaton paar, Austria). The temperature rheological analysis was measured from 25℃ to 80℃. The viscosity test was measured at 0.1% strain and the ω was between 0.6-85 rad/s. The dynamic frequency sweep test was measured at 0.1% strain from 0.1-1Hz. The oscillatory shear rheology test was measured at the frequency of 1 Hz from 0.01-10% strain. The step-strain test was measured at low strain (0.1%) and high strain (300%) at 1 Hz frequency for 700 s. All tests were performed at 25℃ and 37℃ at a concentration of 10 mM.

**Isothermal titration calorimetry (ITC) analysis.** Thermodynamic mechanism was recorded on a NANO ITC (TA, USA). All samples were thoroughly degassed for 30 min before experiments to avoid bubble formation during titration. GA aqueous solution (2 mM) was added into sample cell, and then CuCl_2_ or NCTD aqueous solution (40 mM) was loaded into the syringe. Thereafter, CuCl_2_ or NCTD aqueous solution would inject into the sample cell for 20 times with every injection of 2.5 μL at 298 K. A total of 20 data items were obtained. Accordingly, CuCl_2_ or NCTD aqueous solution titrated into deionized water was used as blank control.

**^1^H-NMR spectrum analysis.** ^1^H-NMR spectrum was recorded on an Avance IIIHD 400 MHz spectrometer (Bruker, America). The lyophilized powder of GA, NCTD and GA-NCTD were dissolved by 1 mL DMSO-*d_6_* and tetramethylsilane was internal standard.


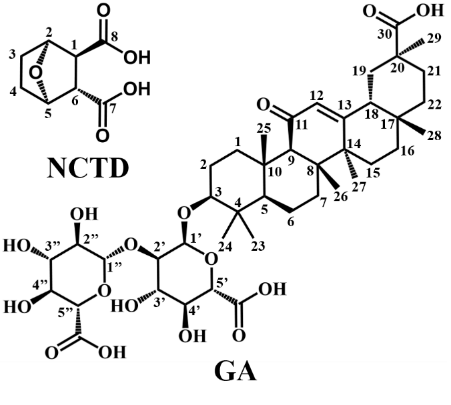


GA ^1^H NMR (400 MHz, DMSO-*d_6_*_,_ δ): 5.39 (s, 1H, 12-H), 4.47 (d, *J* = 7.6 Hz, 1H, 1’-H), 4.34 (d, *J* = 7.6 Hz, 1H, 1’’-H), 3.50-3.00 (m, 14H, 3-H, Glu-H), 2.55 (d, *J* = 13.2 Hz, 1H, 18-H), 2.33 (s, 1H, 9-H), 2.08-0.71 (m, 40H, parent nucleus-H).

NCTD ^1^H NMR (400 MHz, DMSO-*d_6_*_,_ δ): 12.04 (s, 2H, 7-H, 8-H), 4.67 (m, 2H, 2-H, 5-H), 2.90 (s, 2H, 1-H, 6-H), 1.56-1.48 (m, 4H, 3-H, 4-H).

GA-NCTD ^1^H NMR (400 MHz, DMSO-*d_6_*_,_ δ): 5.39 (s, 1H, 12-H, GA), 4.67 (m, 2H, 2-H, 5-H, NCTD), 4.47 (d, *J* = 7.6 Hz, 1H, 1’-H, GA), 4.35 (d, *J* = 7.6 Hz, 1H, 1’’-H, GA), 3.50-3.00 (m, 14H, 3-H, Glu-H, GA), 2.86 (s, 2H, 1-H, 6-H, NCTD), 2.56 (d, *J* = 13.2 Hz, 1H, 18-H, GA), 2.33 (s, 1H, 9-H, GA), 2.08-0.71 (m, 40H, parent nucleus-H, GA), 1.56-1.48 (m, 4H, 3-H, 4-H, NCTD).

**Molecular dynamics (MD) simulation.** The MD simulation was performed by GROMACS 2019.6 software under OPLS-AA force field. Firstly, a simulation box sized 50 × 50 × 50 Å composed of GA, CuCl_2_, NCTD and H_2_O molecules was constructed. Thereafter, the energy was minimized by using the steepest descent method to eliminate unreasonable contact between atoms in the simulation box. And the simulation box was completely relaxed when the maximum force of the system was less than 10 kJ/(mol·nm). Subsequently, a 50 ns sampling simulation was performed using a V-rescale thermostat at 298.15 K and the temperature time constant was set to 2 ps. In the meantime, the long-range electrostatic interaction and van der Waals interaction were calculated by Particle Mesh Ewald (PME) and Cut-off methods respectively. The cut-off radius was set to 1 nm and calculation time step was set to 1 fs, respectively.

**Cell Culture.** The HepG2, Hepa1-6 and MDCK cells were cultured in DMEM supplemented with 10% (v/v) FBS at 37°C (5% CO_2_).

**RNA sequencing (RNA-seq) analysis.** Total RNA was extracted from the tumor tissues using TRIzol® Reagent according to the manufacturer’s instructions (Invitrogen). High-quality RNA (OD 260/280 = 1.8~2.0, OD 260/230 ≥ 2.0, RNA integrity numbers (RIN) ≥ 6.5) was used to construct RNA-seq library, which was prepared following TruSeqTM RNA sample preparation Kit from Illumina (San Diego, CA, USA). The data generated from Illumina platform were used for bioinformatics analysis. All of the analyses were performed using the free online platform of Majorbio Cloud Platform (www.majorbio.com).

**Real-time quantitative polymerase chain reaction (qPCR).** Total RNA was extracted from the tumor tissues using TRIzol® Reagent according to the manufacturer’s instructions (Invitrogen) and then was reverse-transcribed into cDNA using First Strand cDNA Synthesis Kit (Yeasen, Shanghai, China). Real-time qPCR was performed using qPCR SYBR Green Kit (Yeasen, Shanghai, China). Primers for the target genes were synthesized by Beijing Biomed Gene Technology Co., Ltd (Beijing, China) and all sequences were listed in Table S4. Gene expression was normalized and calculated using the 2-ΔΔCт method.

**Immunohistochemistry (IHC) assay and immunofluorescence (IF) staining.** Tumors were embedded in paraffin and fixed with formalin. The tissue sections were deparaffinized in xylene, rehydrated in gradient ethanol and retrieved antigen in antigen retrieval buffer (pH 6.0). Afterwards, using rabbit primary antibodies and goat secondary antibodies according to targeted proteins. In the subsequent IHC assay, the slides were rinsed and counterstained with Mayer's hematoxylin. As for IF staining, slides need to use DAPI counterstain in nucleus and quench spontaneous fluorescence.

**2. Results**

**
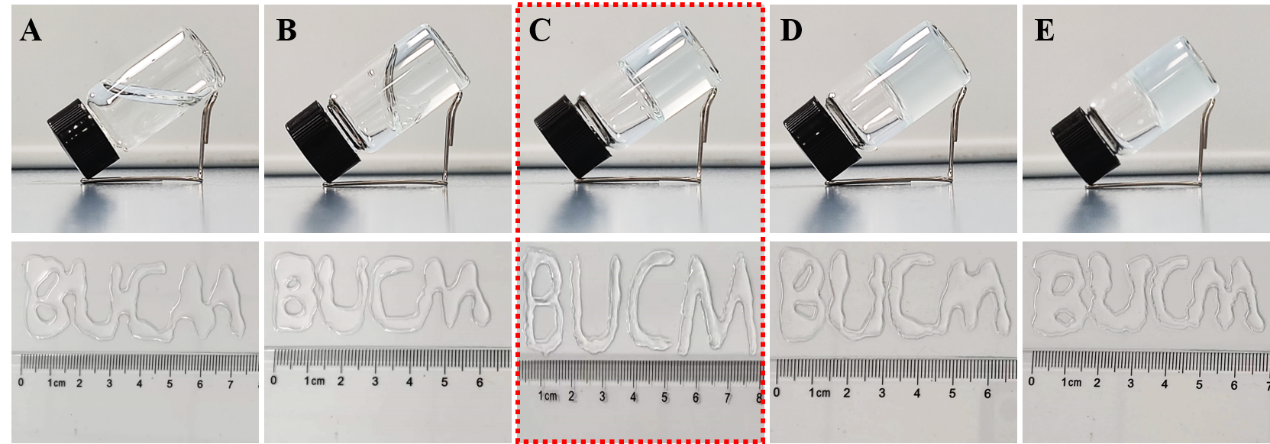
**

**Figure S1**. Photos of GA-Cu hydrogel at a molar ratio of **A** 10:0, **B** 10:2, **C** 10:5, **D** 10:7, **E** 10:10.

**Table S1** Energy changes data for the interactions obtained from ITC.

| Number | Cu^2+^ to GA [*μ*J] | Cu^2+^ to water [*μ*J] | Correction [*μ*J] | NCTD to GA [*μ*J] | NCTD to water [*μ*J] | Correction [*μ*J] |
| --- | --- | --- | --- | --- | --- | --- |
| 1 | -771.5 | -101.6 | -669.9 | -93.74 | -215.5 | 121.8 |
| 2 | -543.4 | -154.1 | -389.3 | -115.6 | -58.48 | -57.15 |
| 3 | -535.7 | -170.6 | -365.1 | -135.2 | -27.28 | -107.9 |
| 4 | -437.1 | -180.6 | -256.4 | -151.7 | -19.69 | -132 |
| 5 | -356.3 | -211.5 | -144.8 | -144.6 | -18.3 | -126.3 |
| 6 | -296.6 | -153.7 | -142.9 | -122 | -18.29 | -103.7 |
| 7 | -289.9 | -164.5 | -125.4 | -97.86 | -14.38 | -83.48 |
| 8 | -332.6 | -182.5 | -150 | -76.37 | -15.08 | -61.29 |
| 9 | -225.9 | -175.2 | -50.67 | -52.93 | -16.77 | -36.16 |
| 10 | -222.3 | -176.9 | -45.39 | -34.65 | -15.16 | -19.49 |
| 11 | -199.1 | -182.8 | -16.26 | -25.86 | -13.98 | -11.88 |
| 12 | -193.3 | -184.1 | -9.176 | -27.97 | -11.69 | -16.29 |
| 13 | -178.7 | -187.9 | 9.185 | -24.22 | -11.73 | -12.49 |
| 14 | -166 | -190.1 | 24.04 | -20.08 | -12.06 | -8.024 |
| 15 | -157.3 | -190.6 | 33.31 | -18.66 | -11.53 | -7.131 |
| 16 | -151.2 | -191.6 | 40.41 | -19.11 | -12.59 | -6.512 |
| 17 | -157.9 | -194 | 36.05 | -17.47 | -14.32 | -3.155 |
| 18 | -153.6 | -191.3 | 37.76 | -17.37 | -13.83 | -3.539 |
| 19 | -157 | -193.4 | 36.46 | -16.11 | -12.4 | -3.714 |
| 20 | -143.7 | -189.3 | 45.6 | -15.32 | -10.68 | -4.639 |

**Table S2** Binding thermodynamics of Cu^2+^ to GA and NCTD to GA.

| No. | Δ*H*  [kJ·mol^-1^] | *-T*Δ*S*  [kJ·mol^-1^] | Δ*G*  [kJ·mol^-1^] | *Kd*  [mol/L] | *Ka*  [L/mol] |
| --- | --- | --- | --- | --- | --- |
| Cu^2+^ to GA | -56.91 | 41.154 | -15.756 | 1.747×10^-3^ | 5.724×10^2^ |
| NCTD to GA | -1.012 | -25.556 | -26.568 | 2.205×10^-5^ | 4.536×10^4^ |


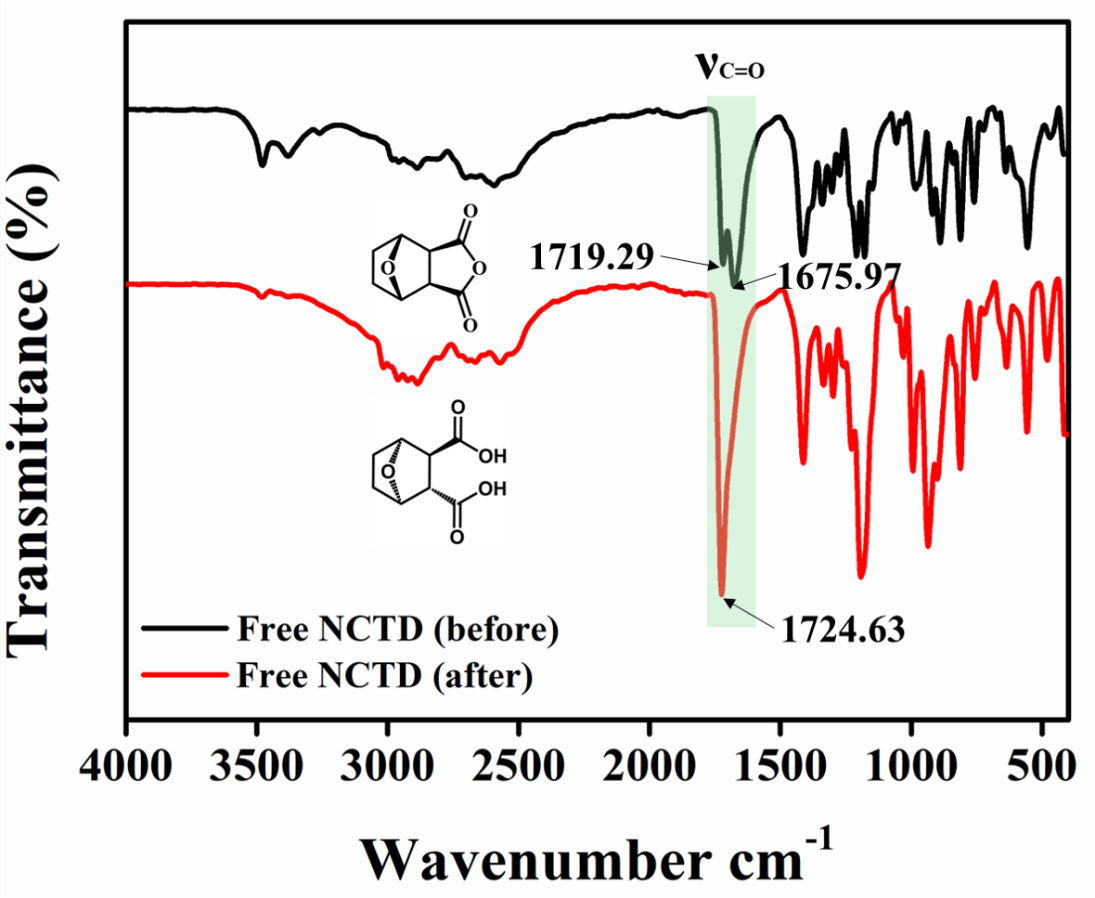


**Figure S2**. FT-IR of free NCTD before and after self-assembly with GA and Cu^2+^.

**Table S3** The number of molecules in simulation box.

| Type | Number |
| --- | --- |
| GA | 20 |
| Cu^2+^ | 10 |
| NCTD | 20 |
| H_2_O | 4000 |





**Figure S3**. Time-dependent changes of SASA in self-assembly process.





**Figure S4**. Release curve of GA in the NCTD Gel in normal and tumor microenvironments. **P* < 0.05, ***P* < 0.01, ****P* < 0.001 (n = 3) versus tumor microenvironments group. ^#^*P* < 0.05, ^##^*P* < 0.01, ^###^*P* < 0.001 (n = 3) versus normal microenvironments group.


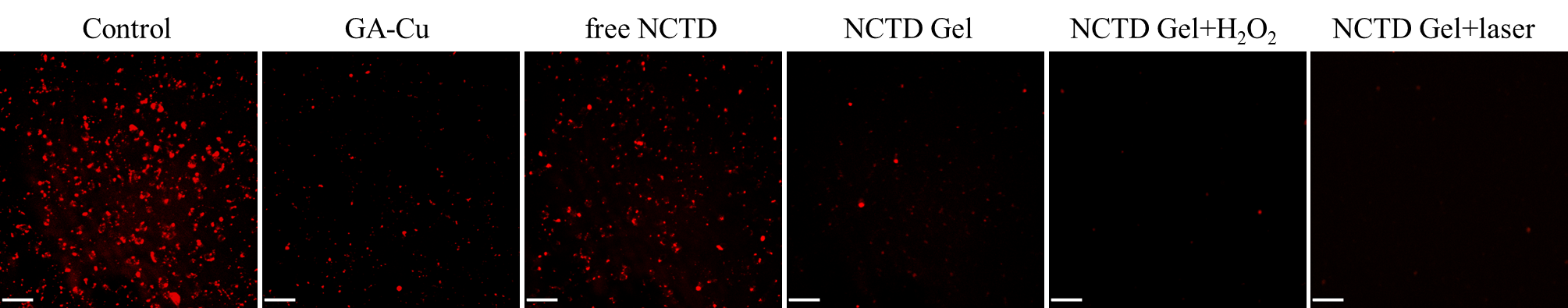


**Figure S5**. RDPP staining with different treatments. Scale bar = 80 μm.


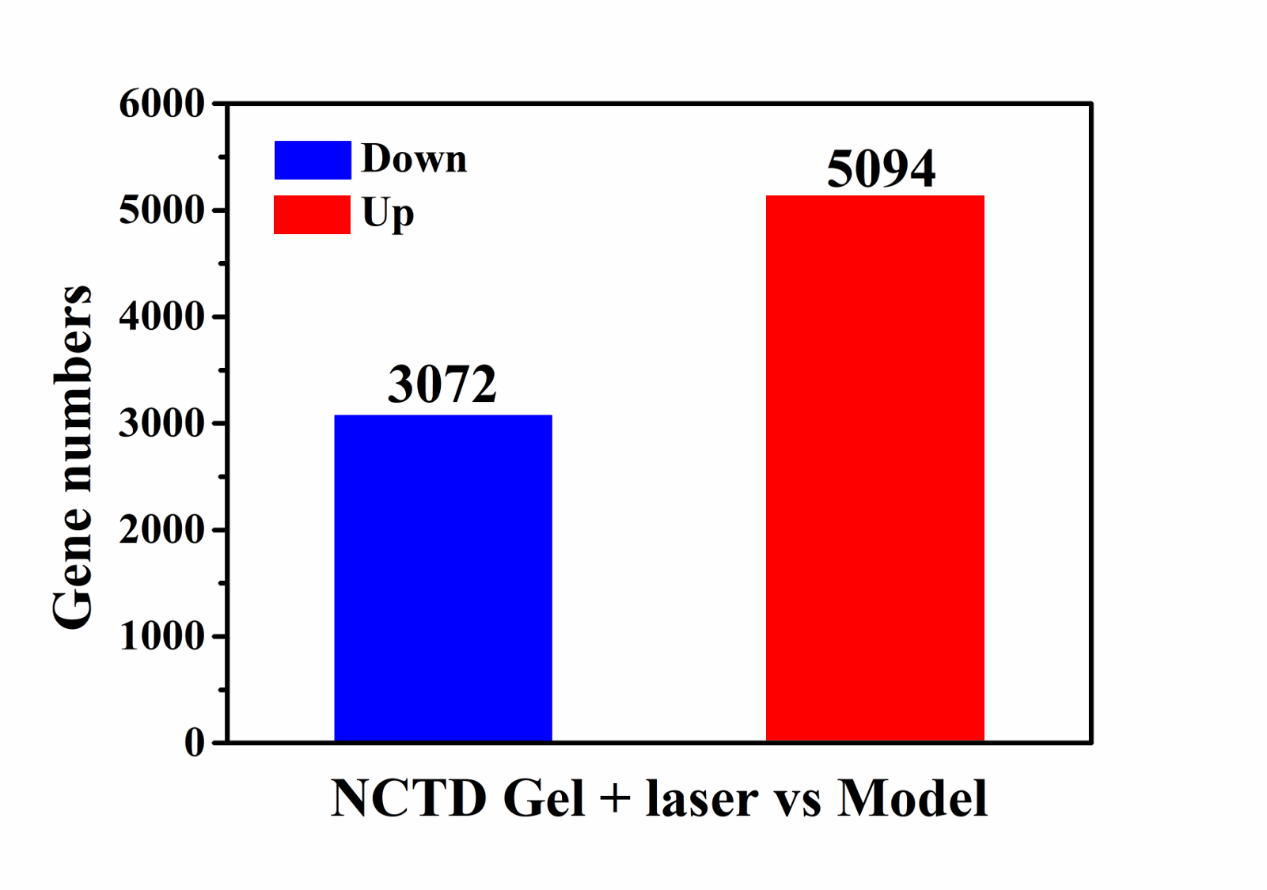


**Figure S6**. Down-regulation and up-regulation of genes in NCTD Gel + laser vs Model.

**Table S4** The Primer sequences used in this study.

| Gene name | Forward primer sequence (5′-3′) | Reverse primer sequence (5′-3′) |
| --- | --- | --- |
| RAPGEF3 | GACCGGAAGTACCACCTTAGG | AGATTCCCACAACTTGGCTCC |
| ICAM-1 | GCCAGCAACTTGGAAATCAGC | GGGGTCGTCACAGAGCTTG |
| CD44 | CTGCCGCTTTGCAGGTGTA | CATTGTGGGCAAGGTGCTATT |
| DLD | GAAATGTCCGAAGTTCGCTTGA | TCAGCTTTCGTAGCAGTGACT |
| DLAT | CGGAACTCCACGAGTGACC | CCCCGCCATACCCTGTAGT |
| PDHA1 | TGGTAGCATCCCGTAATTTTGC | ATTCGGCGTACAGTCTGCATC |
| IL-1β | TTCGACACATGGGATAACGAGG | TTTTTGCTGTGAGTCCCGGAG |
| TNFAIP8L2 | CGGCACTTAGCTTTGGTGAG | GAGTGAAGTCAGGCCCATAGA |
| IL-1RN | CATTGAGCCTCATGCTCTGTT | CGCTGTCTGAGCGGATGAA |


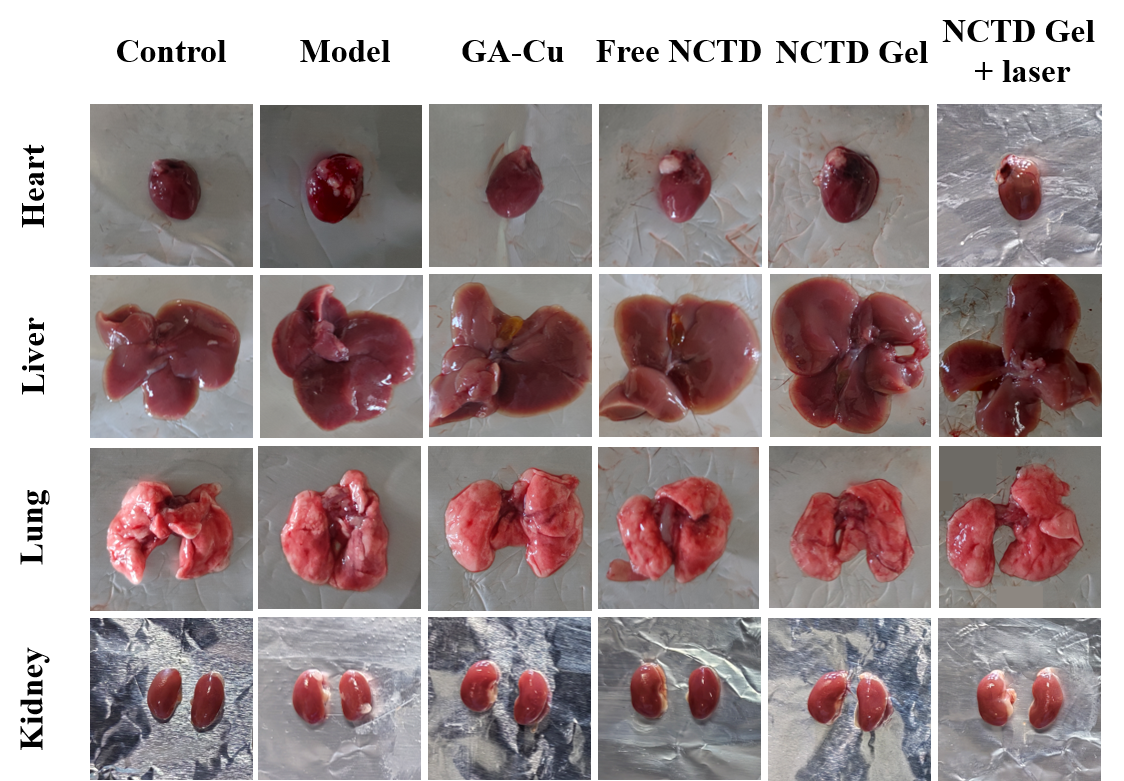


**Figure S7**. Photographs of heart, liver, lung and kidney after various treatments for 13 days.
